# Photobiomodulation in fibroblasts: from light to healing through molecular pathways, omics and artificial intelligence

**DOI:** 10.3389/fbioe.2025.1675619

**Published:** 2025-10-16

**Authors:** Sarassunta Ucci, Eugenio Caradonna, Anna Aliberti, Andrea Cusano

**Affiliations:** ^1^ Optoelectronics Group, Department of Engineering, University of Sannio, Benevento, Italy; ^2^ Centro Regionale Information Communication Technology, CeRICT scrl, Benevento, Italy; ^3^ Department of Clinical Pathology and Pathological Anatomy Italian Diagnostic Center, Via Simone Saint Bon, Milan, Italy

**Keywords:** photobiomodulation, fibroblasts, wound healing, regenerative medicine, omics, artificial intelligence, molecular signaling

## Abstract

Photobiomodulation (PBM), a non-invasive therapy that uses non-ionizing light in the visible to near-infrared spectrum, has proven to be a promising strategy for promoting tissue repair and regeneration. PBM has its roots in heliotherapy and low level light therapies and works by activating specific molecular pathways in the target cells. Among these, fibroblasts play a central role in mediating wound healing responses. This article provides a comprehensive overview of the mechanisms of action of PBM, focusing on the effects on fibroblast biology, including modulation of proliferation, migration and extracellular matrix remodeling. Recent advances in omics technologies and artificial intelligence (AI) provide new tools to unravel the complexity of PBM-induced signaling and optimize therapeutic protocols. By integrating data-rich approaches and systems-level analysis, this work highlights the potential of PBM as a precision-guided regenerative modality and underscores the need for further translational studies to support its clinical application.

## 1 Introduction

In the annals of time, the roots of photobiomodulation (PBM) stretch back to ancient civilizations. The Romans appreciated the benefits of sunbathing, even if they apparently did not attribute any therapeutic properties to it (Sequeira and O’Donovan, 1925). Herodotus, the famous Greek physician from the second century BC, was the father of heliotherapy. His teachings emphasized the usefulness of sun exposure for restoring health (Daniell and Hill, 1991). It was believed that the therapeutic effect was due to the red light and heat of the sun: ultraviolet (UV) rays were not yet known and were discovered only in 1801 (Roelandts, 2002). Phototherapy then became popular as a science thanks to the Danish doctor Niels Finsen, who introduced the use of artificial light produced by a carbon arc to treat lupus vulgaris, a feat that earned him the prestigious Nobel Prize in Medicine in 1903 (Grzybowski and Pietrzak, 2012). The use of artificial lamps gradually changed the application of UV radiation in medicine. By a happy twist of scientific fate, low-level laser therapy (LLLT) was brought to life in the 1960s through the experiments of the Hungarian scientist Endre Mester. His efforts were triggered by the desire to repeat the work of the American luminary Paul McGuff, who had achieved remarkable success in curing malignant tumors in rats with a powerful ruby laser (694 nm) (MgGuff et al., 1965). Although Mester’s laser was considerably weaker than McGuff’s, it was unable to repeat the success of the tumor cure. In this setback, however, Mester made an unexpected discovery: the low-level laser not only stimulated hair growth but also had a profound effect on wound healing. This unforeseen turn of events led to the formulation of a phenomenon now known as PBM, an intricate process involving the activation of biological mechanisms within the targeted tissue. Thus, an unintended discovery gave rise to a thriving field of therapeutic lighting that is shaping the landscape of modern light-based medical interventions (Hamblin, 2016). LLLT is characterized by the fact that light is administered in a dose that is below the threshold of damage and triggers cellular photoactivation: it is a non-thermal process in which chromophores orchestrate a symphony of photophysical and photochemical events at various biological levels. Experimental evidence also reveals that lasers, considered strictly monochromatic and coherent light sources, are not essential for achieving beneficial biological effects; non-coherent light-emitting diodes (LEDs) work in a similar way. The term “low-level laser therapy” (LLLT) has become outdated due to its vagueness and the use of non-laser light sources such as LEDs. The name has been changed to “photobiomodulation” (PBM) to reflect the broader range of applications and avoid confusion (Anders et al., 2015).

### 1.1 The modern PBM

The term PBM define universally the form of light therapy that utilizes non-ionizing forms of light sources including lasers, LEDs, and broadband light, that cover the fascinating spectrum from visible to infrared wavelengths (Avci et al., 2013). Over time, these fundamental discoveries paved the way for modern PBM research, which now focuses on understanding the specific cellular mechanisms and potential therapeutic applications. Scientists are increasingly investigating how PBM can specifically interfere with cellular pathways to influence processes such as tissue repair and regeneration. Building on these findings, this paper will provide a comprehensive overview of the current state of PBM, focusing on its effects on fibroblasts and applications in wound healing. Through a synthesis of studies on molecular signaling pathways activated by PBM in fibroblasts, we will analyze how PBM affects cellular processes involved in tissue repair. To deepen the concept of providing a data-driven, cohesive overview for the advancement of PBM applications in regenerative medicine, the paper may focus on the crucial role of OMICs-based approaches and artificial intelligence (AI) applications in uncovering new insights and identifying research gaps in PBM. This approach emphasizes the need for multilevel, integrative analysis of PBM efficacy in fibroblast-directed wound healing and specifically focuses on advancing the field through data-rich, precision-oriented methods.

### 1.2 Understanding PBM outcomes: the critical roles of photoreceptors, photoacceptors, chromophores and other several factors

The absorption of light energy by living cells depends on the presence of biomolecules that can be excited by light quanta. The essential prerequisite for any photobiological effect is the excitation of these molecules, which can be specialized or unspecialized, by electromagnetic radiation, which leads to a subsequent energy conversion. Photoreceptors are specialized molecules, such as rods and cones, that are sensitive to light and are typically associated with sensory organs such as human eye. Photoreceptor is a broad term for any cellular component that senses and responds to light, and refers primarily to other light-sensitive structures in cells or organisms, even if they are not directly involved in PBM (Bidell et al., 2024). Photoacceptors, on the other hand, are unspecialized molecules that can absorb light but are not part of the light receptor organs. They are ubiquitous molecules in cells or tissues capable of absorbing light and are often integrated into metabolic pathways that have nothing to do with light processing (Karu T., 2000; Gonzalez-Lima, 2011). They are more common and more widespread than photoreceptors and can also be artificially introduced into living systems (Allison and Moghissi, 2013). Photon absorption of certain wavelengths induces a change in the molecular structure of the photoacceptor, subsequently triggering a modification in a signaling cascade as a primary reaction. The adaptations of cellular functions resulting from these initial responses are considered secondary reaction (Kreslavski et al., 2012). Both photoreceptor and photoacceptor molecules share a common feature known as a chromophore, a specific segment responsible for light absorption, that usually consists of organic cofactors or metal ions embedded in a protein structure (Gonzalez-Lima, 2011). PBM has the ability to produce wide-ranging effects on various chromophores throughout the body, depending on the specific wavelength of light used. The key to this fascinating primary mechanism is the conversion of light absorbed by the system into photochemical energy capable of mediating biological effects (Serrage et al., 2019). Different wavelengths of light are absorbed by different molecules, so the modulation of downstream molecular targets can be different. Understanding the mechanism of action of PBM is a challenge due to the existence of different molecules with photoacceptor capabilities. To make matters worse, it is difficult to assign a single molecular target to a specific effective wavelength because the same molecule could be photoacceptor in both the violet-to-blue and red to near-infrared spectral region. This signifies that even when a particular molecule is found to mediate a biological effect for the most part, other possible photoacceptors may also contribute at least in some degree to elicit a particular response (Karu, 1999; Gonzalez-Lima, 2011).

**FIGURE 1:**
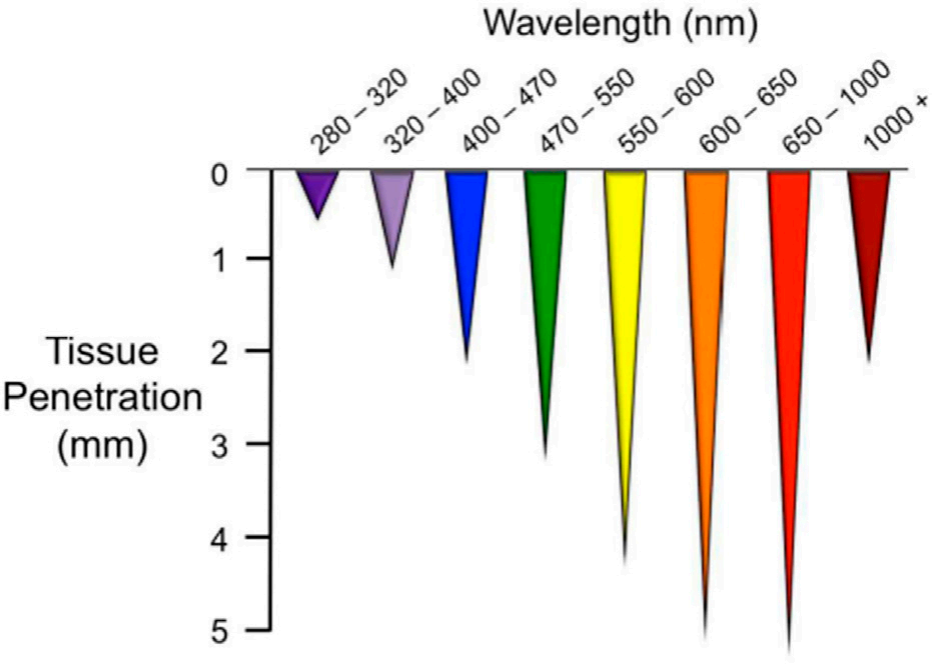
Optical penetration depth (Ruggiero et al., 2016).

Light initiates essential physiological processes vital for human health. It consists of three main spectral regions within the electromagnetic radiation emanating from the sun that reaches Earth: ultraviolet (UV) light, visible light (VL), and infrared light (Sklar et al., 2013). There is increasing evidence that certain wavelengths of electromagnetic radiation, ranging from VL to the near infrared (NIR), have the potential to elicit photophysical and photochemical effects capable of modulating biological processes in human body (Serrage et al., 2019). The efficacy of these outcomes is primarily influenced by the depth of penetration of the utilized wavelength, and these effects may differ based on the specific location of the tissue (Lanzafame, 2020; Chauhan and Gretz, 2021). In general, the longer the wavelength, the greater the penetration depth. In addition, the cellular response may vary depending on the different irradiation parameters. PBM can have extensive effects on a variety of chromophores such as signaling molecules containing flavins and porphyrins, as well as elements of the electron transport chain depending on the wavelength of the light (Serrage et al., 2019; da Silva et al., 2023). Colors are frequently used to denote specific wavelength ranges, such as blue (400–470 nm), green (470–550 nm), yellow (570–595 nm), red (630–700 nm) and NIR light (700–1200 nm) (Barolet, 2008; Ruggiero et al., 2016; Chauhan and Gretz, 2021) which have different penetration depths (Figure 1). Blue light can penetrate as far as 0.07–1 mm (Kumari et al., 2023) in the body and is known for its positive impact on wound healing (Purbhoo-Makan et al., 2022), acne (Diogo et al., 2021; Nakayama et al., 2023) and psoriasis (Pieper et al., 2022). However, it is also associated with adverse effects, such as causing cellular damage through the excessive production of reactive oxygen species (ROS) (Yang et al., 2017; Kumari et al., 2023). Notwithstanding this, there are some studies showing that this increased ROS generation can trigger apoptosis of tumor cells, making blue light irradiation an optimal candidate for an alternative therapy due to its potential anti-tumor effect (Balas et al., 2023; Takeuchi et al., 2023).

Additionally, blue light contributes to photoaging (Pourang et al., 2022; Kumari et al., 2023) and disturbs the circadian rhythm (Cougnard-Gregoire et al., 2023). Blue light acts on various photoacceptors, in particular flavins, porphyrins, nitrosated proteins and opsins (Garza et al., 2017; Serrage et al., 2019). Some of them are also sensitive to green light which effectively penetrates up to a maximum depth of around 2 mm (Barolet, 2008; Ruggiero et al., 2016; Van Tran et al., 2021). Due to its rapid absorption by surface tissues, particularly hemoglobin and water, green light exhibits limited penetration into the deeper tissue layers compared to wavelengths in the red or infrared spectrum (Malthiery et al., 2021). Green light is primarily needed to activate ion channels (Gu et al., 2012; Wang et al., 2014) but clinical applications may be limited due to the lack of penetration depth into tissue. Nevertheless, studies have indicated its effectiveness in improving burn and wound healing processes (Al-Watban et al., 2009; Malthiery et al., 2021). Among the photoacceptors reported to mediate the possible biological effects involving green light include nitric oxide (NO) release from stable NO-carriers such as *S*-nitrosoglutathione and related *S*-nitrosothiols (Sexton et al., 1994). Opsins are a group of cis-retinal dependent G-protein coupled receptors (Wang et al., 2017) that trigger signaling cascades upon distinct wavelength of light. Since most opsins have their absorption peak in the short wave region of the optical spectrum, it has been shown that blue, green, violet, and UV-A regions are most effective in stimulating opsins to activate signal transduction pathways (Terakita and Nagata, 2014; Suh et al., 2020). The yellow light has often been integrated into the green spectrum of the VL in numerous studies and it is assumed that its cellular photoacceptor is mitochondrial protoporphyrin IX (Chauhan and Gretz, 2021). Irradiation with yellow wavelength may be an effective treatment modality for photoaged (Hong et al., 2022) and photodamaged skin (Khoury and Goldman, 2008; Alster and Wanitphakdeedecha, 2009; Chen et al., 2018). However, it is known that red light has the highest penetration depth in VL spectrum, with cytochrome c oxidase, the unit IV of the mitochondrial electron transport chain, being the best researched and validated photoacceptor for this type of light. It contains chromophores that can absorb light, particularly in the red and infrared regions of the electromagnetic spectrum. Such absorption can trigger various cellular responses, potentially influencing cellular activities and increasing energy production (Chauhan and Gretz, 2021; Hamblin and Liebert, 2022; Pchelin et al., 2023). In addition, cytochrome c oxidase is generally believed to act as a primary photoacceptor for light also in the NIR region of the electromagnetic spectrum, but while red light is used to treat superficial tissue, NIR wavelengths are used to affect deeper layers due to the lower light scattering (Pchelin et al., 2023). The effect of NIR irradiation is analogous to that of red light improving the bioavailability of NO through several mechanisms of action (Pchelin et al., 2023), increasing signal transduction accompanied by a temporary rise in ROS, cellular energy availability and calcium (Ca^2+^) levels (Migliario et al., 2018). Ca^2+^ ions play a central role in shaping almost all aspects of cellular activity, but their critical importance in the nervous system is particularly noteworthy. Changes in the concentrations of intracellular calcium ions serve as mediators for signal transduction pathways that control the regulation of neuronal functions (Sharma et al., 2011). It is known that Ca^2+^ membrane transporters are not only found in the plasma membrane of cells (Borrachero-Conejo et al., 2020), but also in the intracellular Ca^2+^ reservoirs of the endoplasmic reticulum (ER) and mitochondria (Contreras et al., 2010). Consequentially, the increase in the intracellular Са^2+^ concentration can be caused by the exogenous influx of Са^2+^ into the cells via cell membrane depolarization and its release from the intracellular organelles (Golovynska et al., 2021). Therefore, PBM can theoretically modulate the Ca^2+^ uptake of the cell via an indirect activation of calcium fluxes, by ROS or adenosine triphosphate (ATP) generation (Bathini et al., 2022), or a direct stimulation of the Ca^2+^ transporters with or without a heating up of microscopic regions of water in the plasma membrane cation channels belonging to the transient receptor potential (TRP) superfamily (Amaroli et al., 2019; Borrachero-Conejo et al., 2020; Golovynska et al., 2021). In PBM, although wavelength is a primary factor, several other variables contribute to its application, including fluence (Huang et al., 2009), polarization (Tripodi et al., 2020), and pulse structure (Priyadarshi et al., 2023). PBM exhibits dose-dependent biological effects, with stimulatory changes observed at low to medium doses, while inhibitory effects are seen at high doses (Zein et al., 2018). Total fluence and exposure time are likely to be interrelated factors influencing the efficacy of PBM. Therefore, understanding and optimizing these parameters are critical to achieving the desired results in PBM applications (Tripodi et al., 2021; Samuel Enwemeka, 2024).

## 2 Methods

This review aims to evaluate and elucidate the existing evidence regarding the efficacy of PBM on fibroblast cells. Specifically, it will undertake a comprehensive analysis of the molecular signaling pathways modulated by PBM to provide a mechanistic understanding of its biological effects. Furthermore, this survey will provide a basis for integrating omics technologies and artificial intelligence methodologies in future investigations, thereby facilitating a more holistic and predictive approach to PBM research with a view towards the assessment of the system biology that lie behind PBM. A comprehensive literature review was conducted using Google Scholar^©^, PubMed^©^, Web of Science^©^, ScienceDirect^©^ and Scopus ^©^databases to identify relevant publications. The search encompassed all studies published up to and including 2024, with a historical perspective extending back to 1925 for publications pertaining to the origins of PBM. The search strategy employed the following keywords, which were queried within the title and/or abstract fields: “Photobiomodulation,” “Low-level light therapy,” “Low-level laser therapy,” “Wound healing and fibroblasts,” “Effects of PBM on fibroblasts,” “Molecular pathways of PBM in fibroblasts,” “omics of PBM in fibroblasts,” and “Systems biology and artificial intelligence in PBM in fibroblasts. Inclusion criteria were defined to focus on *in vitro* studies involving human and animal cell lines, as well as review articles and comparative analyses. Exclusion criteria were not defined, allowing for a broad capture of relevant data. Furthermore, a manual review of the reference lists of retrieved articles was performed to identify additional pertinent studies not initially captured by the database searches. A total of 141 articles were selected based on the aforementioned criteria. The extracted data were subsequently categorized and analyzed across distinct thematic sections to facilitate a systematic synthesis of the findings.

## 3 Wound healing and fibroblast

In 1858, the German pathologist Rudolf Virchow first characterized fibroblasts as “Spindelzelle des Bindegewebes,” translating to “spindle-shaped cells of connective tissue,” a term derived purely from their morphology as observed microscopically. It was not until 1895 that Ernst Ziegler introduced the term “fibroblast” to specifically denote the cells responsible for the deposition of new connective tissue during wound healing processes (Plikus et al., 2021). Fibroblasts are a type of connective tissue cell that play a crucial role in maintaining the structural framework of tissues and organs in the body. They are the most abundant cell type in connective tissue and are responsible for the synthesis and secretion of extracellular matrix (ECM) components, which include collagen, elastin and other proteins (Plikus et al., 2021). Fibroblasts play a central role in the intricate process of wound healing (Tracy et al., 2016) in which they migrate to the affected site in response to injury or tissue damage, proliferate and contribute to the production of collagen facilitating the formation of scar tissue (Darby and Desmoulière, 2020). Beyond their role in wound healing, fibroblasts are integral to tissue repair and remodeling, helping to ensure the proper functioning of organs and structures. They interact with immune cells and various other cell types, contributing to the overall tissue response to injury or inflammation (Davidson et al., 2021). Fibroblasts exhibit considerable heterogeneity: they are found in different tissues of the body, e.g., skin, connective tissue and organs, and different subtypes perform specialized functions depending on the specific tissue environment (Lynch and Watt, 2018). In addition to their physiological roles, fibroblasts can also be involved in pathological conditions, such as fibrosis, which can lead to excessive collagen deposition and tissue scarring (Talbott et al., 2022).

**FIGURE 2:**
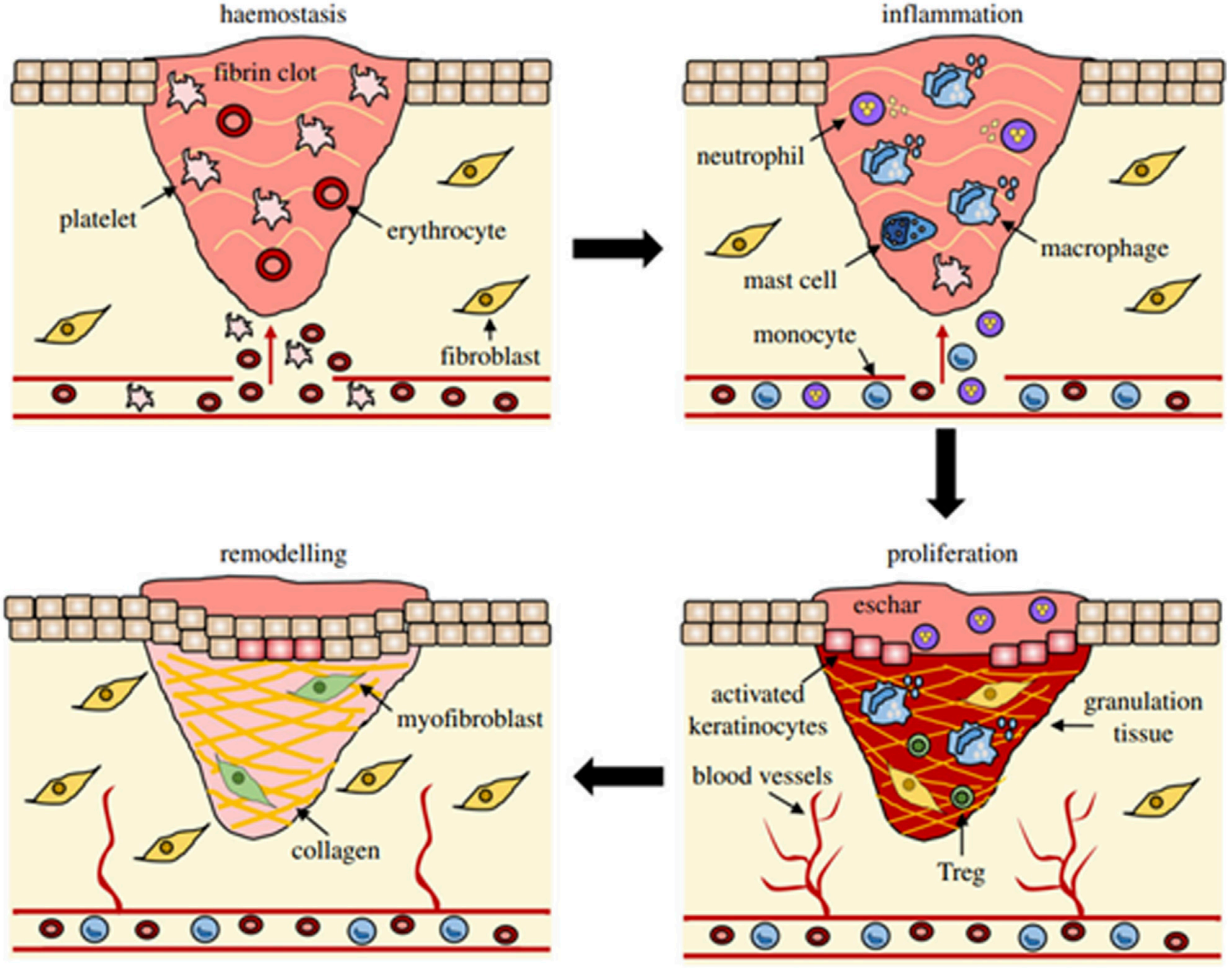
Overview of wound healing stages and their principal cellular elements (Wilkinson and Hardman, 2023).

A comprehensive understanding of the multifaceted functions of fibroblasts is crucial to gain insight into tissue development, repair and disease associated with connective tissue. Wound healing is a complex process involving various cellular mechanisms (Hernández-Bule et al., 2024) that work together to restore the integrity of the tissue: haemostasis, inflammation, proliferation, and remodeling (Wilkinson and Hardman, 2023) (Figure 2). Wound healing begins with haemostasis, where a blood clot forms to prevent excessive bleeding after an injury. The platelets adhere to the blood vessel wall, are activated by thrombin and release molecules that enhance clotting and seal the wound to protect it from bacteria. Inflammation often involves the production of specific cytokines by smooth muscle cells (SMCs) and endothelial cells (ECs). These cytokines play a critical role in promoting cellular proliferation. This response is tightly regulated by a network of signaling pathways and transcription factors. SMCs and ECs proliferate in response to platelet-derived growth factor (PDGF) and facilitate the repair of the vessel wall. Inflammation is a critical response to tissue injury, functioning to eliminate debris and prevent infection. This process involves an initial influx of neutrophils, followed by the arrival of monocytes, which subsequently differentiate into tissue macrophages. During this phase, pro-inflammatory cytokines and growth factors such as TNF-α, IL-1β, IL-6, PDGF, VEGF, GM-CSF are also released to stimulate the next phases of healing (Wilkinson and Hardman, 2023). Fibroblasts come into play during the proliferation phase, in which they play an important role in the organization of tissue repair. Fibroblasts are the main producers of collagen, the predominant protein in connective tissue. Collagen fibers provide tensile strength to the wound, facilitating its closure and preventing further injury. Fibroblasts secrete various growth factors and cytokines that regulate the proliferation and activity of other cell types involved in wound healing, such as endothelial cells and immune cells. These molecules help coordinate the complex cellular interactions required for effective tissue repair. Even more they contribute to the formation of granulation tissue, a temporary scaffold of newly formed blood vessels and connective tissue that fills the wound bed (Bainbridge, 2013). This scaffold serves as a framework for the final phase of healing, the remodeling one, which is characterized by the maturation and reorganization of the newly formed tissue. Remodeling of the ECM is central for wound healing and ranges from the formation of fibrin clots to the development of a mature scar rich in type I collagen (Diller and Tabor, 2022). Fibroblasts ensure the replacement of the ECM and the formation of collagen fibrils through the transition from type III to type I collagen, even if they never fully restore the integrity of the pre-injury tissue. Myofibroblasts, that are specialized contractile fibroblasts stimulated by transforming growth factor-beta (TGF-β), express alpha-smooth muscle actin (α-SMA) filaments allowing them to generate strong contractile forces (Darby et al., 2014). They are particularly important in wound healing for their ability to contract wounds, leading to wound closure and scar formation. Dermal fibroblasts are crucial for wound healing, and their dysfunction is linked to impaired wound healing in diabetes (Yu et al., 2017). Ultimately, the wound healing response ends with the detachment of key cellular components, leaving a mature scar. A deviation from the usual wound healing process can lead to abnormal scarring and a chronic condition that is more susceptible to infection. During this process, fibroblasts undergo a temporary phase of senescence, during which they secrete a specific molecular pattern known as senescence-associated secretory phenotype (SASP). SASP is characterized by the secretion of cytokines/chemokines, growth factors, proteases, pro-inflammatory lipids (oxylipins), small extracellular vesicles and free non-coding nucleic acids (Wang et al., 2024). Under these conditions, this transient phenotypic phase facilitates regulated inflammation and matrix remodeling, thereby mitigating excessive fibrosis (Demaria et al., 2014; Wilkinson and Hardman, 2020). The accumulation of senescent cells with age increases the possibility of chronic wounds. The intricate process is compromised in chronic wounds such as diabetic wounds, vascular ulcers, and pressure ulcers. In diabetic wounds, fibroblasts exhibit impaired migration, reduced secretion of VEGF, and an abnormal response to hypoxia (Lerman et al., 2003). Further diabetic fibroblasts experience alterations in several cytokines pathways as the P13K/Akt, p38MAPK and the IL-1β signaling (Dai et al., 2021; Li et al., 2025). In a diabetic hyperglycemic environment, the increase in oxidative stress and the accumulation of advanced glycation end products sustain SASP, which contributes to the persistence of inflammation and proteolysis, thereby hindering the deposition of a functional matrix (Berlanga-Acosta et al., 2020). Chronic SASP can also be induced by mechanical stress and infections (Wang and Shi, 2020; Reyes et al., 2023). Chronic leg ulcers are characterized by fibroblast senescence, which impairs healing through a prolonged, non-resolving inflammatory phase, the presence of contaminating bacterial populations, and elevated levels of oxidative stress (Wall et al., 2008). Collectively SASP of fibroblasts emerges as a fundamental determinant of both physiological and pathological process of wound repair. These chronic wounds not only have an impact on patients' daily lives and health, but also put a strain on healthcare budgets worldwide and require specialized and intensive treatments. For clinicians and researchers alike, ensuring successful and world-class wound healing is a significant financial challenge. Therefore, the development of better and more creative approaches to skin wound healing is of great medical importance to the healthcare system worldwide. With recent strides in science, technology, and precision medicine, a plethora of emerging and inventive methodologies such as 3D bioprinting, cold plasma therapy, platelet-rich plasma treatments, and ECM-based approaches show promising potential for enhancing the efficacy of wound healing (Kolimi et al., 2022). Nevertheless, the application of one of these methods often does not lead to completely satisfactory results in individual cases, so that numerous wounds are resistant to all available techniques. An alternative approach that shows promise in achieving these goals is the use of PBM either independently or in conjunction with other modalities (Kuffler, 2016). Recent studies show that PBM reduces oxidative stress in diabetic and wounded fibroblasts by modulating the AKT/FOXO1 signaling axis and enhancing endogenous antioxidant defenses (e.g., SOD, CAT, HMOX1) (Rajendran et al., 2021); restores ROS homeostasis, limits inflammatory mediators (NF-κB, TNF-α, IL-1β), and promotes fibroblast proliferation under hyperglycemic stress (Chen et al., 2021); accelerates wound closure and epithelialization in diabetic animal models with improved redox balance (Karkada et al., 2022); and stimulates key regenerative signaling pathways (FGF2, miR-21) to support ECM remodeling and resolution of inflammation (Amini et al., 2023).

## 4 The effects of PBM on fibroblasts: molecular pathways

**FIGURE 3:**
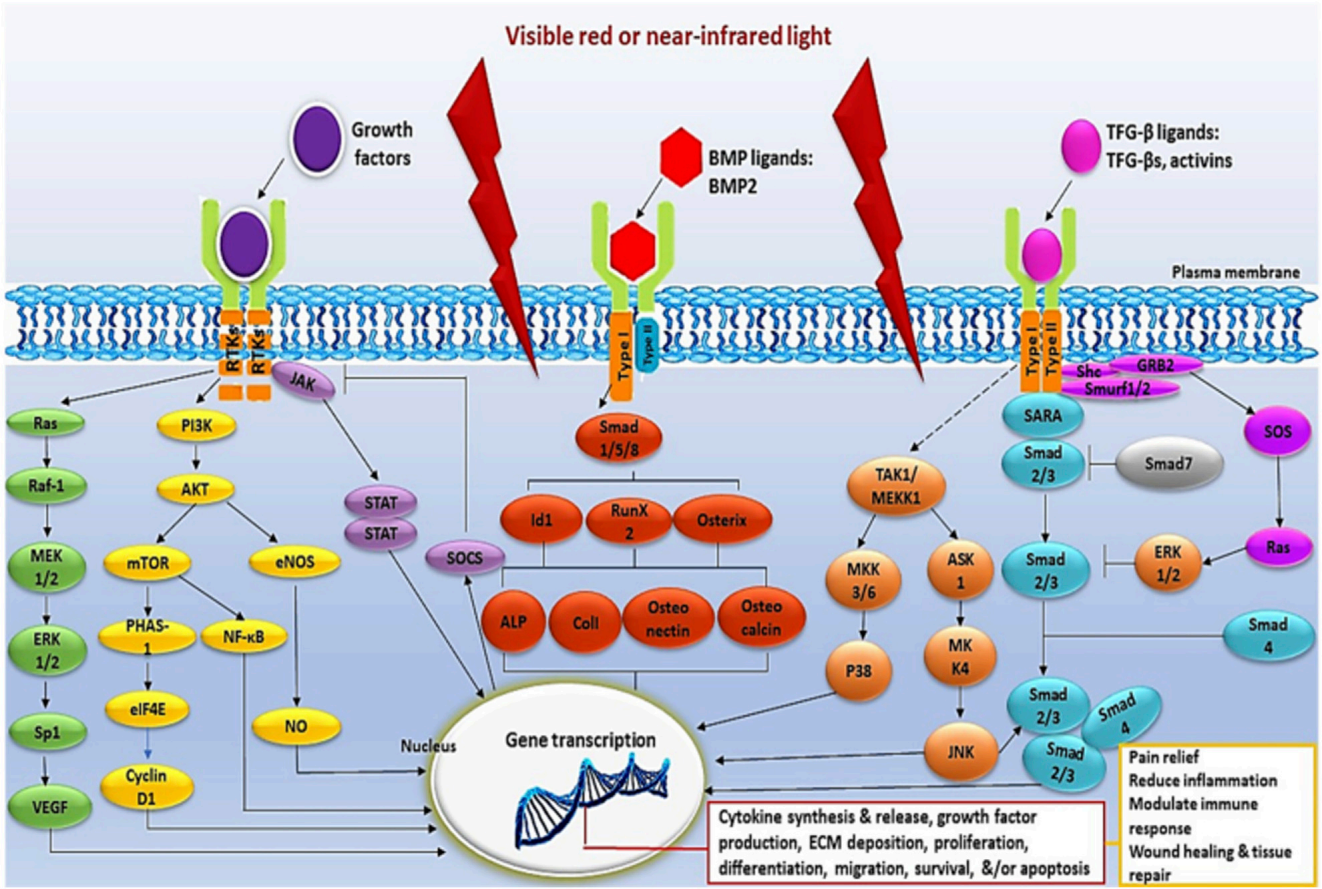
Illustrative schematic of intracellular signaling pathways triggered by PBM (Leyane et al., 2021).

In normal wound healing, fibroblasts alternate between inactive and active states to support tissue repair by secreting growth factors, cytokines and ECM components that are critical for wound closure. However, in diabetic patients, persistent hyperglycemia and metabolic memory disrupt fibroblast behavior and response to stimuli, resulting in impaired phenotype switching (Voza et al., 2024). In poorly healing diabetic wounds, a change in the cellular and molecular signals that are essential for the normal wound healing process is observed. PBM promotes tissue regeneration and speeds up wound repair in various medical conditions, including hard-to-heal diabetic wounds, by reducing inflammation, decreasing cellular apoptosis, and enhancing cellular functions like viability, proliferation, differentiation, metabolism, migration, ECM production, and the release of growth factors, cytokines and proteins primarily through the use of red and NIR light (Kasowanjete et al., 2022; Mgwenya et al., 2024). Growth factors can have endocrine, paracrine, autocrine or intracrine effects by binding to cell surface receptors, activating signaling pathways and regulating gene and protein expression (Leyane et al., 2021) (Figure 3).

### 4.1 Growth factors release

Delayed wound healing is linked to impaired cell function, which leads to reduced migration, proliferation and synthesis of growth factors and collagen. Growth factors, including members of the fibroblast growth factor (FGF) family, play a critical role in the regulation of wound healing. FGF is involved in signal transduction pathways such as Ras/MAPK, phospholipase C (PLC), signal transducer and activator of transcription (STAT) and protein kinase B (AKT). The Ras/MAPK pathway, a well-characterized signal transduction pathway, transmits extracellular signals to the nucleus to activate genes for cell growth, division and differentiation to promote wound healing (Kasowanjete et al., 2022). Kasowanjete et al. (2023) found that irradiating diabetic wounded cells *in vitro* with PBM at 660 nm and 5 J/cm^2^ enhanced cell viability and proliferation. Additionally, irradiated diabetic wounded cell models showed a significant increase in migration rate-almost 3.5 folds (45%)- 24 h post-irradiation. This was determined by measuring the distance between the wound edges and calculating the percentage of cell migration. The improvement was attributed to increased levels of basic fibroblast growth factor (bFGF) and the subsequent activation of the Ras/MAPK signaling pathway (Kasowanjete et al., 2023). Various studies indicate that PBM accelerates wound healing by stimulating fibroblast proliferation. Saygun et al. (2008) discovered that PBM at 685 nm and 830 nm statistically significantly increased the production of growth factors such as bFGF by 6.3-fold (84%) and insulin-like growth factor-1 (IGF-1) by 1.5 fold (34%) compared to the control group in human gingival fibroblasts (hGF) without causing cell damage (cell viability was 109%). They compared the effects of a single and a double dose of laser irradiation (2 J/cm^2^) and observed increased fibroblast proliferation in both groups compared to the control group (Saygun et al., 2008). Furthermore, PBM was shown to have greater efficacy when human skin fibroblasts (HSF) were cultured under high glucose conditions during laser treatment. Esmaeelinejad and Bayat (2013) found that PBM at 632.8 nm and 1 J/cm^2^ stimulated the release of bFGF from HSF that were initially cultured in high glucose media for one or 2 weeks and transitioned to physiological levels during laser treatment with an increase of approximately 2-fold. This study emphasises the importance of maintaining glycemic control during laser treatment for HSF initially cultured under high glucose conditions (Esmaeelinejad and Bayat, 2013). Rahbar Layegh et al. (2020) investigated the effects of PBM on primary human dermal fibroblasts derived from both normal (HDFs) and diabetic (DHDFs) donors. Irradiation at 632.8 nm and 0.5 J/cm^2^ was used to treat diabetic fibroblasts (LT-DHDFs). The analysis revealed that LT-DHDFs exhibited significantly increased secretion of key proteins involved in various cellular processes important for wound healing, including proliferation, migration and inflammatory response. In particular, seven cytokines/growth factors, including BDNF, eotaxin-3 (CCL26), FGF6, FGF7 (KGF), fractalkines (CX3CL1), Fit3 ligand (FT3LG) and GCP2 (CXCL6), showed a more than a two-fold increase in secretion in LT-DHDFs compared to untreated DHDFs, highlighting the potential of PBM to improve cell function impaired by diabetes. Not only is this therapy promising in alleviating the cellular deficits associated with diabetes, but it also underscores its role in promoting wound healing by modulating critical signal transduction pathways (Rahbar Layegh et al., 2020). Damante et al. (2009) observed that human gingival fibroblasts, when exposed to infrared laser at 780 nm with doses of 3 J/cm^2^ and 5 J/cm^2^, exhibited a 1.49-fold increase in bFGF release compared to non-irradiated cells (Damante et al., 2009). There are different opinions as to which wavelength is best suited to achieve positive effects on diabetic fibroblasts. Houreld and Abrahamse (2008) investigated the optimal laser parameters for the healing of diabetic wounds *in vitro*. Fibroblast cells were irradiated with 5 or 16 J/cm^2^ at 632.8 nm, 830 nm or 1,064 nm. The results showed that irradiation with 5 J/cm^2^ at 632.8 nm led to complete wound closure, increased cell viability and enhanced bFGF expression compared to diabetic wounded un-irradiated cells. At 830 nm, partial closure and increased bFGF expression were observed, while at 1,064 nm, incomplete closure with increased apoptosis was observed. Higher doses (16 J/cm^2^) at each wavelength resulted in incomplete occlusion, increased apoptosis and reduced bFGF expression. These results emphasise the wavelength- and dose-dependent responses of diabetically injured cells to laser therapy, with the best results obtained at 5 J/cm^2^ and 632.8 nm (Houreld and Abrahamse, 2008) (Table 1).

**TABLE 1:** PBM and Growth Factors release in fibroblasts.

Cells	λ (nm)	Fluence (J/cm^2^)	Model	Statistical analysis	Outreads	References
Human skin fibroblast cells, namely, normal (N), wounded (W), diabetic (D), and diabetic wounded (DW)	660	5	*In vitro*	Experiments: n = 3, assays in duplicate (averaged) Data: Mean ± standard error (SE) Controls: Non-irradiated (0 J/cm^2^) Stats: Student’s t-test; one-way ANOVA + Dunnett’s *post hoc*	- PBM increased bFGF levels and FGFR activation - Enhanced phosphorylation of Ras, MEK1/2, and MAPK was observed - PBM at 660 nm improved fibroblast viability (p < 0.01), proliferation (p = 0.001) and migration (45%) - The study concludes that PBM at 660 nm promotes *in vitro* diabetic wound healing via the bFGF-activated Ras/MAPK pathway	Kasowanjete et al. (2023)
Human gingival fibroblasts (hGF)	685,830	2	*In vitro*	Software: SPSS 10.0 (SPSS, Chicago, IL, USA) Test: Mann–Whitney U test Significance: p ≤ 0.05	- Irradiated groups showed increased proliferation and viability - Single-dose group: significant increase in bFGF (84%) and IGF-1 (34%), but IGFBP3 increase was not significant - Double-dose group: significant increase in all parameters	Saygun et al. (2008)
Human skin fibroblasts (HSFs)	632.8	1	*In vitro*	Software: SPSS v16 (SPSS Inc.). Parametric tests: Student’s t-test, ANOVA. Nonparametric test: Mann–Whitney U test Significance: p < 0.05 Data format: Mean ± standard deviation	- LLLT with doses of 0.5, 1, and 2 J/cm^2^ significantly stimulated IL-6 release in HSFs cultured in high glucose medium - LLLT with 1 J/cm^2^ induced bFGF release from HSFs cultured in high glucose for 1 or 2 weeks (p < 0.05) with an increase of approximately 2-fold - LLLT was more effective in releasing IL-6 and bFGF when HSFs were cultured in physiological glucose concentration medium	Esmaeelinejad and Bayat (2013)
Primary human dermal fibroblasts harvested from normal and diabetic donors	632,8	0.5	*In vitro*	Experiments: n = 3 Software: SPSS v23; GraphPad Prism 8 Normality test: Kolmogorov–Smirnov (α = 5%) Tests Live/dead → Kruskal–Wallis Normal data → Student’s t-test (2 groups), One-way ANOVA (≥3 groups) Data: Mean ± SD. Significance: p < 0.05	−20 cytokines/growth factors were secreted by DHDFs, with no factor showing more than a two-fold increase compared to HDFs - Seven cytokines/growth factors (BDNF, Eotaxin-3, FGF6, FGF7, Fractalkine, Fit3-ligand, GCP2) showed more than a two-fold increase in LT-DHDFs compared to DHDFs	Rahbar Layegh et al. (2020)
Cell line originating from human gingival tissue	780–660	3–5	*In vitro*	Experiments: n = 3 Data: KGF and bFGF (pg/mL), mean ± SEM Stats: ANOVA + Tukey’s test Significance: p ≤ 0.05	- Infra-red laser treatment significantly increased bFGF release (1.49 fold) compared to other groups - KGF release was consistent across all groups - The elevated bFGF production could be a key mechanism by which infra-red laser promotes wound healing	Damante et al. (2009)
Diabetic-induced wounded human skin fibroblast cells	632.8, 830, or 1,064 nm	5–16	*In vitro*	Experiments: n = 4; tests in duplicate (averaged) Software: SigmaPlot v8.0 Test: One-tailed Student’s t-test Significance: p < 0.05	- Incomplete wound closure, higher bFGF expression with 830 nm (p < 0.000) - Incomplete wound closure, increased apoptosis with 1064 nm (p < 0.000) - Incomplete wound closure, increased apoptosis, decreased bFGF expression with 16 J/cm^2^ at all wavelengths - Diabetic cells responded most effectively to 5 J/cm^2^ at 632.8 nm: complete wound closure, increased cell viability (p = 0.001), and bFGF expression (p = 0.002)	Houreld and Abrahamse (2008)

### 4.2 TGF-β/Smad signaling

In diabetic conditions, growth factors that are important for wound healing are underexpressed (Thomson et al., 2006). It has been shown that PBM can modulate the production and release of factors such as TGF-β1 (Table 2) in a controlled self-limiting manner modality because both ROS and TGF-β1 in excessive amounts are potently deleterious (Ponnusamy et al., 2020). TGF-β together with its receptors (TGF-βRs) and Smad constitute the TGF-β/Smad signaling pathway (Shi and Massagué, 2003) which can facilitate collagen synthesis, skin remodeling, and improve wound healing in fibroblast cells (Zhong et al., 2011). TGF-β molecule represents a family of growth factors in which there are three mammalian isoforms: TGF-β1, TGF-β2 and TGF-β3. TGF-β signaling controls a diverse set of cellular processes: it induces the recruitment of inflammatory cells to the injury site in the early stages of the healing process, which later participate in a negative feedback loop via the release of superoxide from macrophages. The regulation of TGF-β1 in the later stages of the healing process remains a critical issue that needs to be better understood (Pakyari et al., 2013). This has been demonstrated in human skin fibroblast cells where a wavelength of 660 nm and a radiation intensity of 5 J/cm^2^ had increased in cell viability in all irradiated cell models at 24 h post irradiation. Notably, cell viability in irradiated diabetic wounds was 1.5 times higher than in non-irradiated wounds, without any significant effect on the expression on the expression of TGF-β1, pTGF-β1R1, and p-Smad2/3 (Mokoena et al., 2020). However, irradiation at 830 nm using the same fluence of 5 J/cm^2^ significantly increased TGF-β1 levels in diabetic wounded cells at 48 and 72 h post irradiation by almost 1.1 fold. This increase in TGF-β1 contributed to enhanced cell viability and migration, which was coherently significant at 48 h by almost 1.5-fold In addition, irradiation with 830 nm at 5 J/cm^2^ resulted in a remarkable increase in cell proliferation during S-phase compared to non-irradiated controls by almost 1.5-fold at 48 h, supporting the successful healing of diabetic wounds (Oyebode and Houreld, 2022). The cell’s ability to repair every day and traumatic injuries is essential for maintaining tissue integrity. A scratch assay is performed by growing the cells to confluence and creating a “wound” (cell-free zone) on the cell layer into which the cells can migrate. PBM activation of the TGF-β signaling which promotes burn wound epithelial migration was demonstrated on human dermal fibroblast (HOF) (Khan et al., 2021). PBM treatments at 810 nm with a higher dose of 15 J/cm^2^ significantly promoted fibroblast migration The authors further confirmed that these effects are mediated through PBM-activated TGF-β by employing the SB431542 inhibitor for pre-incubation, which effectively nullified the pro-migratory responses in fibroblasts at both three and 15 J/cm^2^ at 810 nm (Khan et al., 2021). Dang et al. (2010) compared the irradiation of cultured human skin fibroblasts with two types of wavelengths (532 nm and 1.064 nm) at the same fluence (1.5 J/cm^2^) and evaluated the mRNA levels of procollagen, matrix metalloproteinases (MMPs), tissue inhibitors of metalloproteinases (TIMPs), heat shock protein 70 (Hsp70), interleukin-6 (IL-6) and TGF-β by RT-PCR 24 and 48 h after irradiation. Based on the study, it was concluded that the 1.064 nm laser significantly increased TGF-β expression after 48 h of irradiation and was generally more effective in promoting positive molecular activities compared to the visible 532 nm laser (Dang et al., 2010).

**TABLE 2:** PBM and TGF-β/Smad signaling in fibroblasts.

Cells	λ (nm)	Fluence (J/cm^2^)	Model	Statistical analysis	Outreads	References
Human skin fibroblast cells. Normal (N), Normal Wounded (NW), Diabetic (D), Diabetic Wounded (DW)	660	5	*In vitro*	Experiments: n = 3 Assays: done in duplicate; mean of duplicates used Statistical analysis software: SigmaPlot v13.0 Tests used: one-tailed Student’s t-test and one-way ANOVA Significance threshold: P < 0.05 Data presentation: mean ± SEM (positive and negative error bars)	-Significant increase (p < 0.01) in cell viability in all irradiated cell models at 24 h. Light at 660 nm and 5 J/cm^2^ significantly enhanced cell viability showing 1.5 times greater cell viability than non-irradiated diabetic wounds at 24 h (p < 0.001)- No real significant changes in TGF-β1, pTGF-β1R1, or p-Smad2/3 - As incubation time post-irradiation increased Thy-1 (CD90) decreased, EDA-FN and α-SMA increased in wounded models	Mokoena et al. (2020)
Human skin fibroblast cell line: Normal (N), Normal Wounded (NW), Diabetic (D), Diabetic Wounded (DW) and Hypoxic Diabetic Wounded (HDW)	830	5	*In vitro*	Experiments: n = 3 Data: mean ± SEM Software: SPSS v27 Statistical tests Student’s t-test (control vs. experiment) One-way ANOVA (between cell groups) Two-way ANOVA (irradiation vs. non-irradiation) Significance: p < 0.05	- Higher presence of TGF-β1 (1.1 fold) in the culture media of irradiated cells - Significant increase (1.5 fold) in cellular migration in wounded models after 48 h post irradiation compared to controls - Irradiation at 830 nm and 5 J/cm^2^ increased S-phase cell proliferation by nearly 1.5-fold at 48 h (p < 0.01), supporting improved diabetic wound healing - Reduction in pTGF-βR1 levels -Slight presence of p-Smad2/3 in the irradiated cells	Oyebode and Houreld (2022)
Human Ovarian Fibroblasts (HOF)	810	3–15	*In vitro*	Experiments: n = 2–3 Each experiment: included replicates	-PBM treatments significantly enhanced fibroblast migration (p < 0.05) at a higher dose of 15 J/cm^2^ - Effects were mediated via PBM-activated TGF-β - Pre-incubation with SB431542 abolished the pro-migratory responses in fibroblasts (p < 0.05) both at 3 and 15 J/cm^2^ at 810 nm	Khan et al. (2021)
Human skin fibroblasts	532; 1064	1,5	*In vitro*	Software: SPSS 15.0 Data format: Mean ± SD Test used: Student’s t-test Significance level: p < 0.05	−532-nm and 1,064-nm lasers stimulate procollagen and TIMP gene expression while inhibiting MMP expression -The 1,064-nm laser may enhance collagen synthesis through TGF-β upregulation (p < 0.05); increased Hsp70 and IL-6 levels may also contribute to improved collagen production	Dang et al. (2010)

### 4.3 JAK/STAT signaling

Epidermal growth factor (EGF) is essential for wound healing as it promotes cell proliferation and migration. EGF binds to its receptor (EGFR), leading to receptor dimerization and tyrosine autophosphorylation, which initiates the JAK/STAT pathway. Four JAK (Janus kinase) and seven STAT (Signal Transducer and Activator of Transcription) family members are involved in this signaling pathway. The JAK/STAT signaling pathway mediates various cellular reactions and regulates the transcription of genes that are crucial for cellular migration, proliferation and differentiation. Cells such as fibroblasts, endothelial cells, keratinocytes and macrophages are involved in JAK/STAT signaling. Pathological conditions, including inflammatory diseases and the development of chronic wounds, can result from a disruption of this regulation (Jere et al., 2017). Jere et al. (2018) demonstrated that diabetic wounded fibroblast cells exposed to PBM at a wavelength of 660 nm and fluence of 5 J/cm^2^ and incubated for 48 h exhibited increased cell migration, proliferation and viability, accompanied by enhanced EGF secretion, which subsequently activated the JAK/STAT signaling pathway. The same group demonstrated that PBM treatment (660 nm, 5 J/cm^2^) upregulated genes involved in the JAK/STAT signaling pathway more strongly in diabetic wounded WS1 cells than in non-diabetic wounded WS1 cells. INSR and SOCS4 genes related to the JAK/STAT signaling pathway were upregulated in irradiated wounded WS1 cells compared to 21 genes upregulated (including JAK3, TYK2, and STAT1, STAT2, STAT3, STAT5A, STAT6) in irradiated diabetic wounded WS1 cells. This increased regulation could be due to the fact that the cells are more stressed by the high glucose content and PBM is likely to be more effective in stressed cells to improve diabetic wound healing (Jere et al., 2020). A similar modulation of expression of genes related to JAK/STAT signaling was achieved by Leyane et al. (2024) by irradiated wounded and diabetic wounded fibroblast cells with light at a wavelength of 830 nm and a fluence of 5 J/cm^2^. In the wounded cell model, five (CEBPB, FCGR1A, GATA3, JUN, SOCS3) of the 84 genes related to the JAK/STAT signaling pathway were significantly upregulated and 4 (CSF1R, ISG15, MYC, SMAD2) downregulated, while in diabetic wounded cells, 6 genes (EGFR, F2, IL2RG, MPL, MYC, OAS1) were downregulated (Table 3).

**TABLE 3:** PBM and JAK/STAT signaling in fibroblasts.

Cells	λ (nm)	Fluence (J/cm^2^)	Model	Statistical analysis	Outreads	References
WS1 human skin fibroblast cells. Four models, namely, normal (N), wounded (W), diabetic (D) and diabetic wounded (DW) were used	660	5	*In vitro*	Experiments: n = 4, assays in duplicate (average used) Software: SigmaPlot 13.0 Tests: One-tailed Student’s t-test, One-Way ANOVA Significance: P < 0.05 Data presentation: Mean ± SEM (with error bars)	- Irradiated diabetic wounded cells showed a significant increase in EGF (p < 0.001) and activation of its receptor (p-EGFR) - Activation of JAK/STAT pathway (p < 0.001) (p-JAK2, p-STAT1, p-STAT5) was observed and stimulated cell proliferation (p < 0.001), migration (p < 0.05) and viability (p P < 0.001) - PBM at 660 nm and 5 J/cm^2^ modulated cellular autocrine signaling, particularly the EGF/EGFR loop	Jere et al. (2018)
WS1 human fibroblasts (wounded and diabetic wounded)	660	5	*In vitro*	Experiments: n = 3 Analysis: RT^2^ Profiler PCR Array, ΔΔCT method Normalization: Average of 5 housekeeping genes Fold change: >1 = upregulation, <1 = downregulation (vs controls) Significance: P ≤ 0.05	- PBM (600 nm, 5 J/cm^2^) regulated JAK/STAT pathway genes in both wounded and diabetic wounded WS1 cells - In wounded cells, 2 genes (INSR and SOCS4) were upregulated - In diabetic wounded cells, 21 genes were upregulated, including JAK3, TYK2, and STAT1, STAT2, STAT3, STAT5A, STAT6 - The upregulation of these genes activated JAK/STAT signaling, enhancing wound healing through processes like migration and proliferation	Jere et al. (2020)
Human fibroblast cell culture: wounded (W) and diabetic wounded (DW)	830	5	*In vitro*	Experiments: n = 3 Software: Qiagen GeneGlobe Analysis: ΔΔCT method (2^(ΔΔCT)) Normalization: 5 reference genes (ACTB, B2M, GAPDH, HPRT1, RPLP0) Significance: p ≤ 0.05	- PBM at 830 nm and 5 J/cm^2^ altered JAK/STAT pathway genes in both wounded and diabetic wounded cells - In wounded cells, 5 genes (CEBPB, FCGR1A, GATA3, JUN, SOCS3) were upregulated, and 4 downregulated (CSF1R, ISG15, MYC, SMAD2) - In diabetic wounded cells, 6 genes (EGFR, F2, IL2RG, MPL, MYC, OAS1) were downregulated	Leyane et al. (2024)

### 4.4 PI3K/Akt signaling

The phosphoinositide 3-kinase (PI3K)/protein kinase B (AKT) signaling pathway is an important intracellular signaling pathway involved in the regulation of various cellular processes such as growth, proliferation, survival, and metabolism. PI3K catalyzes the phosphorylation of phosphoinositides in the cell membrane in response to extracellular signals such as growth factors or insulin. Upon activation, PI3K produces phosphatidylinositol-3,4,5-trisphosphate (PIP3), which recruits AKT to the cell membrane, which is activated by phosphorylation at residues Thr308 and Ser473 (Manning and Cantley, 2007). Oxidative stress induced by high glucose blocks the AKT pathway, and in diabetics, downregulation of the PI3K/AKT pathway is widespread and leads to poor wound healing and tissue regeneration (Rajendran et al., 2021). Transcription factors, including the Forkhead family of transcription factors class ‘O' (FOXO), play a role in orchestrating events critical for normal wound healing. FOXO1, a member of the FOXO subgroup, regulates the insulin/PI3K/AKT signaling pathway and is implicated in wound healing, although its specific role in this process remains incompletely understood (Rajendran et al., 2019). PBM treatment with light of 660 and 830 nm (5 J/cm^2^) reversed the increased oxidative stress in diabetic and diabetic-wounded fibroblast cells by decreasing FOXO1 levels and increasing the concentration of the enzymatic antioxidants superoxide dismutase (SOD), catalase (CAT) and heme oxygenase (HMOX1), probably via activation of the AKT signaling pathway. Under high glucose conditions, PBM enhanced cell viability and migration by increasing AKT and antioxidant levels while reducing FOXO1 expression, with 660 nm being slightly more effective than 830 nm in modulating the AKT/FOXO1 pathway—improving viability by 8.5% compared to approximately 6% with 830 nm (Rajendran et al., 2021). The same group investigated a novel mechanism of PBM at 660 and 830 nm on human WS1 fibroblast and adipose-derived stem cell (ADSC) co-cultures. In the study, the samples were divided into four groups: normal (N), wounded (W), diabetic (D), and diabetic wounded (DW). At a dose of 5 J/cm^2^, both wavelengths effectively increased PI3K and AKT levels, improving wound healing in an *in vitro* scratch wound model with diabetic ADSC fibroblast co-cultures (Rajendran and Houreld, 2022). Another study confirmed that a wavelength of 660 nm (5 J/cm^2^) enhances therapeutic activities in diabetic wound healing by activating the PI3K/AKT signaling pathway with an increase of approximately 16% and stimulating downstream signaling proteins (mTOR, GSK3β) in fibroblast cells *in vitro* (Jere et al., 2022). Zhang et al. (2009) demonstrated that PBM promotes cell proliferation via the PI3K/Akt signaling pathway by testing irradiation with 632.8 nm at different fluences (0.2, 0.4, 0.8 and 1.2 J/cm^2^) on african green monkey SV40-transformed kidney fibroblast cell line (COS-7). They found that PBM significantly enhanced cell proliferation in a dose-dependent manner, with 1.2 J/cm^2^ being the optimal dose. PBM induced continuous Akt activation, which was completely inhibited by wortmannin, a PI3K inhibitor, suggesting that the effect of PBM on Akt activation is PI3K-dependent (Zhang et al., 2009). A recent work conducted by Monteiro et al. (2024) demonstrated that was possible to observe a higher relative expression of PI3K-pathway and differentiation after PBM (660 nm, 3 j/cm^2^) (Table 4).

**TABLE 4:** PBM and PI3K/AKT in fibroblasts.

Cells	λ (nm)	Fluence (J/cm^2^)	Model	Statistical analysis	Outreads	References
WS1 human skin fibroblast cells, namely, normal (N), wounded (W), diabetic (D) and diabetic wounded (DW)	660; 830	5	*In vitro*	Experiments: n = 3, in duplicate Data: Mean ± SEM Software: SigmaPlot 13.0 Tests: Student’s t-test, One-Way ANOVA Significance: *p ≤ 0.05, **p ≤ 0.01, ***p ≤ 0.001	- PBM reversed oxidative stress in diabetic fibroblasts: it reduced FOXO1 and increased SOD, CAT, and HMOX1 levels - Effect linked to AKT pathway activation - PBM enhanced diabetic wound healing via FOXO1 inhibition −660 nm wavelength was slightly more effective than 830 nm in regulating the AKT/FOXO1 pathway improving viability by 8.5% compared to approximately 6% with 830 nm (p < 0.01)	Rajendran et al. (2021)
Human WS1 Fibroblast cells and adipose derived stem cell (ADSC) were co-cultured in a 1:1 ratio and were divided into four groups; normal (N), wounded (W), diabetic (D) and diabetic wounded (DW)	660; 830	5	*In vitro*	Experiments: n = 3, in duplicate Data: Mean ± SEM Software: SigmaPlot 13.0 Tests: Student’s t-test, One-Way ANOVA Significance: *p ≤ 0.05, **p ≤ 0.01, ***p ≤ 0.001	- Both 660 nm and 830 nm wavelengths effectively increased PI3K and AKT levels (p < 0.05) - These effects improved wound healing in an *in vitro* scratch wound model with diabetic ADSC fibroblast co-cultures	Rajendran and Houreld (2022)
WS1 human fibroblasts, namely, wounded (W), diabetic wounded (DW) and hypoxic diabetic wounded (HDW)	660	5	*In vitro*	Experiments: n = 2, 3 (average used) Data: Mean ± SEM Software: SigmaPlot 13.0 Tests: Student’s t-test, One-Way ANOVA Significance: *p ≤ 0.05, **p ≤ 0.01, ***p ≤ 0.001	- PBM at 660 nm (5 J/cm^2^) enhances diabetic wound healing: increased activation of PI3K/AKT (16%) and downstream proteins (mTOR, GSK3β) observed - Therapeutic effects of PBM are linked to activation of the PI3K/AKT signaling pathway - The results suggest that activation of GSK3β and mTOR further supports the healing process in stressed cells	Jere et al. (2022)
African green monkey SV40-transformed kidney fibroblast cell line	632.8	0, 0.2, 0.4, 0.8, and 1.2	*In vitro*	Experiments: n = 3 Data: Mean ± SEM Test: Student’s paired t-test Significance: p < 0.05	- PBM activated Akt in a PI3K-dependent manner (blocked by wortmannin) - Src family involvement in Akt activation was shown by partial inhibition with PP1 (Src inhibitor) - PBM promoted cell proliferation (p < 0.01) via PI3K/Akt; inhibited by PI3K inhibitor -PI3K/Akt pathway regulates PBM-induced cell proliferation in a time- and dose-dependent manner	Zhang et al. (2009)
Primary culture of Human Gingival Fibroblasts (HGFs)	660	3	*In vitro*	Experiments: n = 3 Software: GraphPad Prism 9.3.0 Normality test: Shapiro-Wilk Tests: One-way ANOVA + Dunnett’s and Tukey’s post-tests; Fisher’s exact test Data: Mean ± SD (bar graphs) Significance: p < 0.05	- PBM increased activation of PI3K/AKT, mTOR, and GSK3β signaling proteins - The results suggest PBM promotes therapeutic effects by activating PI3K/AKT and downstream proteins, aiding diabetic wound healing	Monteiro et al. (2024)

## 5 Omics, system biology and AI

**FIGURE 4:**
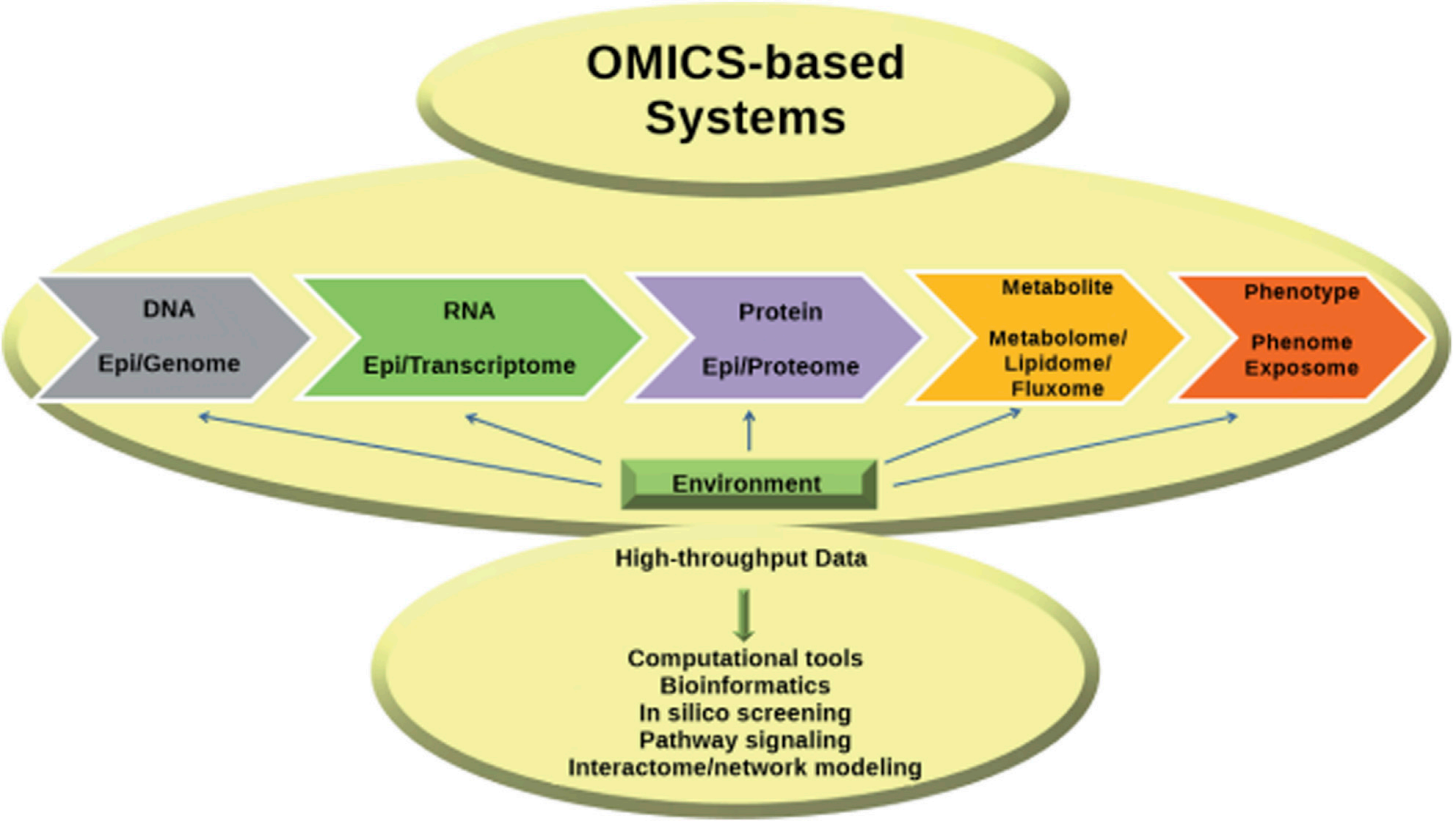
Advances in omics technologies have deepened insights into the central dogma, with bioinformatics essential for data integration, paving the way for systems biology (Nalbantoglu and Karadag, 2019).

A key concept in molecular biology is the transmission of information from DNA to RNA to proteins, ultimately leading to the production of metabolites. The subject has been extensively investigated utilizing high-throughput technologies across the domains of genomics, transcriptomics, proteomics, and metabolomics. The huge amounts of data generated require bioinformatics for analysis and integration and form the basis of systems biology, which attempts to understand the entire system and its functions by taking into account molecular interactions at different levels and translating them into defined networks (Aizat et al., 2018; Nalbantoglu and Karadag, 2019) (Figure 4).

Currently, numerous variables may influence the application of PBM, yet the mechanisms involved remain incompletely understood. Consequently, further research is essential to elucidate the therapeutic effects of polarized light. Evidence from *in vivo* studies has demonstrated that PBM can modulate wound healing, with reports describing beneficial in impaired healing contexts in animal (Byrnes et al., 2004; Priyadarshi et al., 2023) and humans (Saied et al., 2011; Mathur et al., 2017; Raizman and Gavish, 2020). These findings provide a rationale for investigating the molecular and cellular mechanisms underlying PBM. In this regard, PBM in fibroblasts has been increasingly studied using omic technologies, which provide comprehensive insights into the molecular and cellular mechanisms involved. However, it is important to critically consider the inherent limitations of these research models. *In vitro* studies are particularly sensitive to factors such as culture conditions, fibroblast type (e.g., papillary, reticular, or dermal), donor age, sex, and the site of tissue collection, all of which can strongly influence experimental outcomes and affect their reproducibility and translational relevance (Sorrell and Caplan, 2004). Similarly, rodent wound models do not fully replicate human wound healing (Galiano et al., 2004; Gurtner et al., 2011), though they remain valuable for mechanistic studies, pathway validation, and hypothesis generation. These considerations underline the need for critical interpretation of *in vitro* and animal data, and for the integration of multiple approaches to ensure robust and biologically relevant conclusions.

### 5.1 Genomic

Genomic studies can reveal how PBM influences gene expression in fibroblasts. For example, PBM can modulate the expression of genes involved in cell proliferation, migration, and collagen synthesis, which are crucial for wound healing. There are no specific studies on the effects of PBM on fibroblasts in wound healing at the gene level, but Bicknell et al. (2019) investigated the influence of PBM on the microbiome of mice. Mice were irradiated with red (660 nm) or infrared (808 nm) low-level lasers at 10 J/cm^2^ to the abdomen for 2 weeks. Genomic DNA from fecal pellets was pyro sequenced for the 16S rRNA gene, revealing a significant difference in microbial diversity between PBM- and sham-treated mice, an effect that was most pronounced in mice treated with infrared light three times per week and not detectable with a single red light treatment. For example, by day 14, the abundance of the bacterial genus Allobaculum showed a significant increase following infrared PBM, whereas red light PBM had no such effect. This preliminary study suggests that PBM can alter the diversity of the microbiome and increase the number of beneficial bacteria in mice. If confirmed in humans, PBM could be used as an adjunct therapy for obesity, lifestyle-related disorders, cardiovascular disease and neurodegenerative diseases, warranting further clinical investigation (Bicknell et al., 2019).

### 5.2 Transcriptomic

Transcriptomic analyses can reveal changes in mRNA levels in fibroblasts after PBM treatment (Table 5). This helps to understand how PBM regulates gene expression at the transcriptional level and can reveal specific signaling pathways that are activated by PBM (Ayuk et al., 2014; Houreld et al., 2014; Ayuk et al., 2016). In a study conducted by Houreld et al. (2018) the effect of PBM on cell adhesion molecules (CAMs) in diabetic wound healing was investigated using human skin fibroblasts in a diabetic wound model. A diode laser at 660 nm with a fluence of 5 J/cm^2^ was used, and cells were analyzed 48 h after irradiation. RT^2^ Profiler PCR Array showed that PBM modulated the expression of 64 CAM-related genes, with 10 genes upregulated and 25 downregulated. These genes were associated with transmembrane molecules (11 genes (including CD44, ITGA2, and ITGB1) were downregulated, while 5 (such as CDH1, ITGA8, and VCAM1) were upregulated), cell-cell adhesion (CD44, COL6A2, CTNND1 were downregulated and 5 (CDH1, COL11A1, COL14A1, ITGA8 and VCAM1) were upregulated) and cell-matrix adhesion (9 genes were downregulated, including CD44, ITGA3, and ITGB3, whereas 3 (ITGA8, ITGAL, ITGB4) were upregulated. Even more, among other CAMs, 11 genes were downregulated (including COL5A1, FN1, LAMA1) and CNTN1, LAMA3 were upregulated). PBM at 660 nm altered CAM gene expression and improved healing, indicating its potential benefit in combination with other therapies to treat diabetic wounds (Houreld et al., 2018). Zhang et al. (2003) used a cDNA microarray technique to investigate the gene expression profiles of human fibroblasts HS27 irradiated with low-intensity red light (628 nm) at an optimal dose of 0.88 J/cm^2^. They found that irradiation can affect the expression of 111 genes belonging to different 10 functional categories, seven of which are directly or indirectly involved in cell proliferation (Zhang et al., 2003). RNA sequencing (RNA-seq) is a powerful technique used to analyze the transcriptome, the complete set of RNA transcripts produced by the genome under specific circumstances or in a specific cell in order, for example, to quantify the levels of mRNA in a sample, allowing researchers to determine which genes are active, their expression levels, and how they change under different conditions. Tripodi et al., 2023 evaluate transcriptomic changes in human Caucasian fetal foreskin fibroblasts (HFFF2) treated with 0.5 μM hydrogen peroxide (H_2_O_2_) to induce oxidative stress in response to polarized PBM at 670 nm at 1/cm^2^ by RNA seq. A total of 71 differentially expressed genes (DEGs) were described. All DEGs were found in the polarized PBM treated group compared to the control group. The study has shown that PBM has a strong impact on several areas of mitochondrial energy production and signaling pathways associated with wound healing, which opens up many possibilities for further research (Tripodi et al., 2023).

**TABLE 5:** Transcriptomics studies for PBM in fibroblasts.

Cells	λ (nm)	Fluence (J/cm2)	Model	Statistical analysis	Outreads	References
Human skin fibroblasts isolated from a consenting adult undergoing abdominoplasty. Normal Wounded (NW), Diabetic (D),Diabetic Wounded (DW)	660	5	*In vitro*	Experiments: n = 3 Analysis: SABiosciences Excel template Test: Student’s t-test (based on fold change) Significance: p < 0.05	A marked increase in gene expression was observed, with 29 genes upregulated in normal cells, 32 genes upregulated in normal wounded cells and 18 genes upregulated in diabetic wounded cells. Conversely, gene expression was significantly reduced in the following cells: 19 genes in normal cells, 6 genes in normal wounded cells and 31 genes in diabetic wounded cells	Ayuk et al. (2014)
Isolated human skin fibroblast cells. Normal (N-) and diabetic wounded (DW-) cells	830	5	*In* *vitro*	Experiments: n = 3 Analysis: SABiosciences Excel template Method: Relative gene expression (2^^(-ΔΔCt)^) Test: Student’s t-test Significance: p < 0.05	- In N-cells, 28 genes were significantly upregulated and 3 significantly downregulated- In DW-cells, 5 genes were significantly upregulated and 17 significantly downregulated- PBM Stimulated various cell adhesion molecules (CAMs), including cadherins, integrins, selectins and immunoglobulins	Ayuk et al. (2016)
WS1 human skin fibroblasts	660	5	*In vitro*	Experiments: n = 3 Normalization: Average of 5 housekeeping genes Analysis: SABiosciences Excel template Test: Student’s t-test Significance: p < 0.05	−48 h after irradiation at a wavelength of 660 nm with an intensity of 5 J/cm^2^, the expression of 84 genes was analyzed using real-time RT-qPCR in WS1 fibroblast cells. - Of these, 43 genes (related to ECM, Remodeling Enzymes, Cellular Adhesion, Cytoskeleton, Inflammatory Cytokines/Chemokines, Growth Factors, Signal Transduction) showed significant upregulation, while 33 genes (related to ECM, Remodeling Enzymes, Cellular Adhesion, Cytoskeleton, Inflammatory Cytokines/Chemokines, Growth Factors, Signal Transduction) were significantly downregulated	Houreld et al. (2014)
Human skin fibroblasts isolated from a consenting adult undergoing abdominoplasty. Normal Wounded (NW), Diabetic (D), Diabetic Wounded (DW)	660	5	*In vitro*	Experiments: n = 3 Normalization: Average of 5 housekeeping genes Analysis: SABiosciences Excel template Test: Student’s t-test Significance: p < 0.05	- A total of 64 genes were analyzed, with 25 showing significant downregulation and 10 showing significant upregulation- Among transmembrane molecules, 11 genes were downregulated, including CD44, ITGA2, and ITGB1, while 5 were upregulated, such as CDH1, ITGA8, and VCAM1- For cell-cell adhesion molecules, 3 genes were downregulated (CD44, COL6A2, CTNND1) and 5 were upregulated (CDH1, COL11A1, COL14A1, ITGA8 and VCAM1)- Regarding cell-matrix molecules, 9 genes were downregulated, including CD44, ITGA3, and ITGB3, whereas 3 were upregulated (ITGA8, ITGAL, ITGB4)- Lastly, among other CAMs, 11 genes were downregulated (including COL5A1, FN1, LAMA1) and 2 were upregulated (CNTN1, LAMA3)	Houreld et al. (2018)
Normal human fibroblasts of HS27 newborn foreskin	628	0.44, 0.88, 2.00, 4.40, and 8.68	*In vitro*	Experiments: n = 2 Software: ScanAnalyze Threshold: >2-fold (up), <0.5-fold (down) for functional analysis	- A total of 111 genes were found to be regulated by red light irradiation, and these genes were grouped into 10 functional categories - Many of the regulated genes play direct or indirect roles in promoting cell proliferation and inhibiting apoptosis, highlighting the potential of red light to enhance cell growth and survival. - The Key signaling pathways involved are p38 MAPK and PDGF pathways involved in cell growth and repair - Additional gene expression changes were related to antioxidation and to mitochondrial energy metabolism	Zhang et al. (2003)
Human caucasian foetal foreskin fibroblasts	670	1	*In* *vitro*	Experiments: n = 4 DEG Criteria: FDR ≤0.05 Functional Enrichment: STRING-db (GO, KEGG, Reactome pathways) Significance Threshold: p < 0.05	−71 DEGs identified, with 10 upregulated and 61 downregulated genes in polarized PBM group compared to the control group-Most DEGs were related to mitochondria and the extracellular matrix-Gene Ontology analysis revealed significant enrichments in: 95 biological process terms; 18 molecular function terms; 32 cellular component terms ed 21 significantly enriched pathways-Reactome pathway analysis revealed 24 significantly enriched pathways	Tripodi et al. (2023)

### 5.3 Proteomic

Proteomic approaches, such as mass spectrometry, can be used to analyze protein expression and post-translational modifications in fibroblasts after PBM. This can reveal how PBM affects protein levels and activities, including those of growth factors, cytokines, and enzymes involved in extracellular matrix remodeling. Recent advances in imaging mass spectrometry now allow the analysis of unicellular fibroblasts and their ECM in clinically relevant tissue samples. While conventional proteomic methods exist for analyzing individual fibroblasts, further technological advances are needed to fully understand the stromal proteome and apply these assays in the clinical setting (Angel et al., 2021; Ogita et al., 2015) demonstrated that low-level Er:YAG laser irradiation (2.94 μm at 2.11 and 2.61 J/cm^2^) increased the proliferation activity of HGFs by 13%–22% without causing cell damage. Proteomic analysis by Liquid Chromatography - Tandem Mass Spectrometry (LC-MS/MS) identified 59 upregulated proteins 24 h after irradiation. These data are important for the understanding of wound healing phenomena associated with laser therapy and for the development of new therapeutic strategies. The study also showed that the upregulation of galectin-7 could partly explain the increase in cell proliferation (Ogita et al., 2015). There are obviously further proteomic studies on the effects of photobiomodulation on wound healing in models other than fibroblasts. Keshri et al., (2021) compared the effects of pulsed (10 Hz) NIR laser (810 nm) and LED (808 ± 3 nm) treatments on full-thickness third-degree burn wounds in rats. Using comprehensive quantitative label-free global proteomics followed by various validation assays (biophysical, biochemical, molecular, histological and immunohistochemical), they found that both treatments modulated similar biological pathways involved in tissue repair, including those related to neuronal, metabolic, vascular, inflammatory and cell signaling processes. Proteomic analysis identified both known and novel molecules involved in the tissue repair process. The study concluded that both laser and LED treatments at the specified wavelength and in pulsed mode are equally effective in accelerating the healing of full-thickness burn wounds by PBM (Keshri et al., 2021).

### 5.4 Metabolomic

Metabolomic profiling can assess the changes in metabolites within fibroblasts after PBM treatment. This provides insights into the metabolic pathways influenced by PBM and how these changes support cellular functions such as energy production, redox balance, and biosynthesis. The most commonly used techniques for conducting metabolomics studies are mass spectrometry (MS) and nuclear magnetic resonance spectroscopy (NMR) (Moco, 2022). These techniques were used in different experimental model (dos Santos Cardoso, 2021) to compare the cortical and hippocampal metabolic pathways of young (4 months old) and old (20 months old) control rats with those of rats exposed to a transcranial near-infrared laser (810 nm) for 58 consecutive days. Statistical analyses of brain metabolomics data showed that chronic transcranial photobiomodulation significantly improved metabolic pathways in young rats, especially for excitatory neurotransmission and oxidative metabolism. Furthermore, the altered metabolic pathways in older rats are restored to the levels observed in younger rats, particularly in the cerebral cortex. These novel metabolomics results may help to complement other laser-induced neurocognitive, neuroprotective, anti-inflammatory and antioxidant effects described in the literature. Future studies should comprehensively investigate the metabolic and molecular changes using advanced technologies such as NMR-based metabolomics. This approach aims to identify the changes induced by LED therapy, including inhibition, stimulation and metabolic shifts, to better understand the mechanisms and ensure safe use. The gaps in the understanding of light-induced photobiomodulation and the lack of metabolomic studies, especially in fibroblasts, emphasise the urgent need for further research.

### 5.5 System biology and AI

The emergence of various “omics” fields has profoundly changed our understanding of modern biology and led to significant technological advances and scientific breakthroughs. Systems biology emerged from the desire to understand cells holistically and go beyond the study of isolated components. This interdisciplinary approach aims to decipher biological systems by examining the interactions and connections between different components rather than looking at them in isolation. It combines experimental data, computational analysis and mathematical modeling to gain a deep understanding of how biological systems function at different levels of complexity—from molecules and cells to tissues, organs and whole organisms (Aizat et al., 2018). To understand complex biological systems requires the integration of experimental and computational research—in other words a systems biology approach. Computational biology comprises two main branches: knowledge discovery, which uses data mining to uncover hidden patterns from large-scale experimental data and generate hypotheses, and simulation-based analysis, which uses *in silico* experiments to test hypotheses and make predictions that are then validated through *in vitro* and *in vivo* studies (Kriete and Eils, 2005). Future research could use machine learning and optimization algorithms to investigate a broader range of fluence and irradiance parameters (Oyebode et al., 2021). The development of an AI-based systems biology approach to understand the PBM effects of fibroblasts in wound healing represents a promising opportunity to advance therapeutic strategies, particularly for wound healing. The vision could envision the paradigm of Precision Photobiomodulation significantly addressing current challenges, pitfalls and bottlenecks. Currently, the application of PBM in fibroblast research remains largely unexplored, and few comprehensive models exist. One such model (Hsieh et al., 2022) focuses on the effect of PBM on cytochrome c, the main photoacceptor of red and NIR light involved in the mitochondrial electron transport chain. In the study (Hsieh et al., 2022) a mathematical model was proposed to analyze the effect of PBM on NO inhibition in the reduced cyclooxygenase process, taking into account the direct release and reduction of NO, along with its reactions with superoxide radicals. The model tracks the transitions between key states of cytochrome c oxidase (NO-bound, free reduced, and oxidized) and includes the kinetics of NO binding and photodissociation, as well as interactions with reactive oxygen species like superoxide. Simulations accurately reflected experimental oxygen consumption data under various wavelengths, bolstering a mechanism where light-triggered NO dissociation and superoxide-driven NO scavenging boost mitochondrial respiration. This framework offers a molecular-level mechanistic rationale for PBM’s effects and emphasizes how the response varies with wavelength. The model underscores PBM’s capacity to modulate cellular metabolism and energy generation; however, more investigation is necessary to broaden the mechanistic comprehension and its use in wound healing.

## 6 Discussion

Current data on gene expression profiles, CAM regulation, and other molecular factors, including cytokines and growth factors, offer valuable insights for the development of advanced AI models. These models build upon those previously proposed and partially evaluated in the literature. By utilizing these datasets, an AI-driven approach could help to map the networks and pathways that govern fibroblast responses to PBM. To achieve this goal, quantitative gene expression data (upregulated and downregulated genes), cellular pathways and the effects of PBM on specific CAMs, cytokines and growth factors are needed. These aspects need to be linked to both input parameters, such as wavelength, intensity, and fluence, and outcome data, such as observable effects like cell proliferation, migration, collagen production, and wound healing progression. An AI-driven approach could reveal patterns in the data that are difficult to detect using conventional methods, allowing researchers to identify potential biomarkers for effective PBM treatment, predict patient-specific responses and optimize therapy protocols. Despite its immense potential, there are several obstacles to the further development of AI-assisted systems biology for fibroblast PBM, particularly in wound healing. Current datasets are often fragmented and limited in scope. To develop accurate models, data from multiple sources, including gene expression, proteomics and clinical outcomes, need to be integrated. Furthermore, it is essential to consider the complexity of the model, as fibroblast responses to PBM are highly dynamic and context-dependent. AI models must account for the variability in cell types, wound stages, and individual patient characteristics, which presents a significant challenge in developing universal models. Furthermore, AI models need solid experimental validation to ensure that predictions match actual biological outcomes. This requires interdisciplinary collaboration between AI experts and biologists to generate high-quality, reproducible data. Real-time monitoring of fibroblast behavior during PBM treatment would provide continuous input to AI models and allow dynamic adjustments and refinements to the therapeutic approach. By addressing these challenges, the integration of AI with systems biology could significantly improve our understanding of fibroblast behavior under PBM and potentially lead to personalized treatments for chronic wounds. The development of AI-powered models could accelerate the discovery of new biomarkers, optimize the use of PBM in the clinical setting and pave the way for future advanced wound healing therapies. Mechanistic hypotheses generated from *in vitro* omics studies necessitate *in vivo* validation to confirm their biological relevance. The robust integration of *in vitro*, *in vivo*, and computational approaches provides a more comprehensive understanding of PBM effects, thereby enhancing the translatability and reliability of research findings while appropriately acknowledging model-specific limitations.

## 7 Challenge and limitation

Despite its promise, the clinical application of PBM continues to face key challenges. Chief among them are the heterogeneity of irradiation parameters, variability in experimental protocols, and the fragmented nature of data reporting and integration. Overcoming the current barriers requires a decisive shift toward precision PBM—an approach that goes beyond generalized protocols by incorporating patient-specific biological profiles to develop tailored light-based treatments. Such a strategy brings PBM into alignment with the broader framework of personalized medicine, where the aim is to deliver the right treatment, at the right time, to the right patient. To reach this level of therapeutic precision, coordinated efforts are required to standardize experimental conditions, establish interoperable and comprehensive omics datasets, and implement AI-driven models capable of decoding complex dose–response dynamics. By following an integrative and personalized approach, PBM could move beyond its current role as a supportive therapy and establish itself as a core, precision-guided medical intervention—particularly valuable in addressing chronic, diabetic, and therapy-resistant wounds with greater effectiveness and consistency.

## 8 Conclusion

This review offers a comprehensive and up-to-date synthesis of the effects of PBM on fibroblasts, particularly in the context of wound healing. PBM exerts its regenerative effects on fibroblasts through the modulation of several intracellular signaling pathways, each orchestrating key cellular functions essential for wound repair. The Ras/MAPK pathway is prominently activated by PBM via the upregulation of growth factors such as bFGF, resulting in enhanced cell viability, proliferation, and migration. For example, PBM at 660 nm and 5 J/cm^2^ significantly increased fibroblast migration in diabetic wounded models, with a 3.5-fold improvement in motility 24 h post-irradiation, attributed to activation of the Ras/MAPK cascade (Kasowanjete et al., 2023). Similarly, the TGF-β/Smad pathway, which regulates ECM synthesis and myofibroblast differentiation, is influenced by near-infrared PBM. Irradiation at 830 nm and 5 J/cm^2^ promoted TGF-β1 secretion (1.1-fold increase) and significantly enhanced fibroblast migration and proliferation in diabetic and hypoxic conditions (Oyebode and Houreld, 2022). This effect was mechanistically validated by studies demonstrating that inhibition of TGF-β signaling abolished the PBM-induced migration of fibroblasts (Khan et al., 2021). The JAK/STAT pathway is also responsive to PBM stimulation. Red light at 660 nm and 5 J/cm^2^ increased EGF expression and EGFR phosphorylation, which triggered JAK2 and STAT1/5 activation, resulting in improved proliferation and migration, especially in diabetic wounded fibroblasts (Jere et al., 2018; Jere et al., 2020). Moreover, PBM activates the PI3K/Akt signaling axis, a crucial mediator of cell survival and oxidative stress adaptation. PBM (660 or 830 nm, 5 J/cm^2^) increased PI3K and Akt expression while decreasing the levels of FOXO1, a transcription factor linked to cellular stress. These effects were accompanied by an upregulation of antioxidant enzymes (SOD, CAT, HMOX1), leading to enhanced viability in diabetic and hypoxic models (Rajendran et al., 2021; Jere et al., 2022; Rajendran and Houreld, 2022).

Altogether, PBM promotes fibroblast proliferation, migration, ECM production, and differentiation into myofibroblasts by targeting complementary molecular pathways. The most effective biological responses have been consistently reported when using red light (620–700 nm) and near-infrared light (780–850 nm) at fluences of 3–5 J/cm^2^, particularly in diabetic and hypoxic wound models, where endogenous repair mechanisms are compromised (Houreld and Abrahamse, 2008; Jere et al., 2018; Jere et al., 2020; Jere et al., 2022; Rajendran et al., 2019; Mokoena et al., 2020; Khan et al., 2021; Oyebode and Houreld, 2022; Kasowanjete et al., 2023).

The integration of omics technologies—including genomics, transcriptomics, proteomics, and metabolomics—has significantly enriched our understanding of the complex molecular and cellular mechanisms underlying PBM. Coupled with systems biology and artificial intelligence, high-throughput data streams provide a robust framework for predicting individual responses and refining therapeutic strategies with greater precision. However, the full clinical potential of PBM can only be realized through an integrative and personalized approach that combines standardized protocols, interoperable omics datasets, and AI-driven predictive models to develop tailored and truly effective treatments.

## References

[B1] Aizat W. M. Ismail I. Noor N. M. (2018). Recent development in omics studies. Adv. Exp. Med. Biol. 1102, 1–9. 10.1007/978-3-319-98758-3_1 30382565

[B2] Al-Watban F. A. H. Zhang X. Y. Andres B. L. Al-Anize A. (2009). Visible lasers were better than invisible lasers in accelerating burn healing on diabetic rats. Photomed. laser Surg. 27 (2), 269–272. 10.1089/PHO.2008.2310 18707242

[B3] Allison R. R. Moghissi K. (2013). Photodynamic therapy (PDT): PDT mechanisms. Clin. Endosc. 46 (1), 24–29. 10.5946/CE.2013.46.1.24 23422955 PMC3572346

[B4] Alster T. S. Wanitphakdeedecha R. (2009). Improvement of postfractional Laser erythema with light-emitting diode photomodulation. Dermatol. Surg. 35 (5), 813–815. 10.1111/j.1524-4725.2009.01137.x 19397672

[B5] Amaroli A. Ferrando S. Benedicenti S. (2019). Photobiomodulation affects key cellular pathways of all life-forms: considerations on old and new laser light targets and the calcium issue. Photochem. Photobiol. 95 (1), 455–459. 10.1111/PHP.13032 30281800

[B6] Amini A. Ghasemi Moravej F. Mostafavinia A. Ahmadi H. Chien S. Bayat M. (2023). Photobiomodulation therapy improves inflammatory responses by modifying stereological parameters, microRNA-21 and FGF2 expression. J. Lasers Med. Sci. 14, e16. 10.34172/JLMS.2023.16 37583493 PMC10423949

[B7] Anders J. J. Lanzafame R. J. Arany P. R. (2015). Low-level light/laser therapy *versus* photobiomodulation therapy. Photomed. Laser Surg. 33 (4), 183–184. 10.1089/PHO.2015.9848 25844681 PMC4390214

[B8] Angel P. M. Rujchanarong D. Pippin S. Spruill L. Drake R. (2021). Mass spectrometry imaging of fibroblasts: promise and challenge. Expert Rev. Proteomics 18 (6), 423–436. 10.1080/14789450.2021.1941893 34129411 PMC8717608

[B9] Avci P. Gupta A. Sadasivam M. Vecchio D. Pam Z. Pam N. (2013). Low-level laser (light) therapy (LLLT) in skin: stimulating, healing, restoring. Seminars Cutan. Med. Surg. 32 (1), 41–52. 24049929 PMC4126803

[B10] Ayuk S. M. Houreld N. N. Abrahamse H. (2014). Laser irradiation alters the expression profile of genes involved in the extracellular matrix *in vitro* . Int. J. Photoenergy 2014, 1–17. 10.1155/2014/604518

[B11] Ayuk S. M. Abrahamse H. Houreld N. N. (2016). The role of photobiomodulation on gene expression of cell adhesion molecules in diabetic wounded fibroblasts *in vitro* . J. Photochem. Photobiol. B Biol. 161, 368–374. 10.1016/j.jphotobiol.2016.05.027 27295416

[B12] Bainbridge P. (2013). Wound healing and the role of fibroblasts. J. Wound Care 22 (8), 407–412. 10.12968/jowc.2013.22.8.407 23924840

[B13] Balas M. Nistorescu S. Badea M. A. Dinischiotu A. Boni M. Dinache A. (2023). Photodynamic activity of TMPyP4/TiO2 complex under blue light in human melanoma cells: potential for cancer-selective therapy. Pharmaceutics 15 (4), 1194. 10.3390/PHARMACEUTICS15041194 37111678 PMC10144582

[B14] Barolet D. (2008). Light-emitting diodes (LEDs) in dermatology. Seminars Cutan. Med. Surg. 27 (4), 227–238. 10.1016/j.sder.2008.08.003 19150294

[B15] Bathini M. Raghushaker C. R. Mahato K. K. (2022). The molecular mechanisms of action of photobiomodulation against neurodegenerative diseases: a systematic review. Cell. Mol. Neurobiol. 42 (4), 955–971. 10.1007/s10571-020-01016-9 33301129 PMC8942959

[B16] Berlanga-Acosta J. A. Guillén-Nieto G. E. Rodríguez-Rodríguez N. Mendoza-Mari Y. Bringas-Vega M. L. Berlanga-Saez J. O. (2020). Cellular senescence as the pathogenic hub of diabetes-related wound chronicity. Front. Endocrinol. 11, 573032. 10.3389/FENDO.2020.573032 33042026 PMC7525211

[B17] Bicknell B. Liebert A. Johnstone D. Kiat H. (2019). Photobiomodulation of the microbiome: implications for metabolic and inflammatory diseases. Lasers Med. Sci. 34 (2), 317–327. 10.1007/s10103-018-2594-6 30074108

[B18] Bidell D. Feige N. D. Triphan T. Müller C. Pauls D. Helfrich-Förster C. (2024). Photoreceptors for immediate effects of light on circadian behavior. iScience 27 (6), 109819. 10.1016/J.ISCI.2024.109819 38770135 PMC11103378

[B19] Borrachero-Conejo A. I. Adams W. R. Saracino E. Mola M. G. Wang M. Posati T. (2020). Stimulation of water and calcium dynamics in astrocytes with pulsed infrared light. FASEB J. 34 (5), 6539–6553. 10.1096/fj.201903049R 32202681

[B20] Byrnes K. R. Barna L. Chenault V. M. Waynant R. W. Ilev I. K. Longo L. (2004). Photobiomodulation improves cutaneous wound healing in an animal model of type II diabetes. Photomed. Laser Surg. 22 (4), 281–290. 10.1089/PHO.2004.22.281 15345169

[B21] Chauhan A. Gretz N. (2021). Role of visible light on skin melanocytes: a systematic review. Photochem. Photobiol. 97 (5), 911–915. 10.1111/php.13454 33987856

[B22] Chen L. Xu Z. Jiang M. Zhang C. Wang X. Xiang L. (2018). Light-emitting diode 585 nm photomodulation inhibiting melanin synthesis and inducing autophagy in human melanocytes. J. Dermatological Sci. 89 (1), 11–18. 10.1016/j.jdermsci.2017.10.001 29065997

[B23] Chen H. Tu M. Shi J. Wang Y. Hou Z. Wang J. (2021). Effect of photobiomodulation on CCC-ESF reactive oxygen species steady-state in high glucose mediums. Lasers Med. Sci. 36 (3), 555–562. 10.1007/s10103-020-03057-4 32643032

[B24] Contreras L. Drago I. Zampese E. Pozzan T. (2010). Mitochondria: the calcium connection. Biochimica Biophysica Acta - Bioenergetics 1797 (6–7), 607–618. 10.1016/j.bbabio.2010.05.005 20470749

[B25] Cougnard-Gregoire A. Merle B. M. J. Aslam T. Seddon J. M. Aknin I. Klaver C. C. W. (2023). Blue light exposure: ocular hazards and prevention—A narrative review. Ophthalmol. Ther. 2023 12 (2), 755–788. 10.1007/S40123-023-00675-3 36808601 PMC9938358

[B26] da Silva T. G. Ribeiro R. S. Mencalha A. L. de Souza Fonseca A. (2023). Photobiomodulation at molecular, cellular, and systemic levels. Lasers Med. Sci. 38 (1), 136–11. 10.1007/s10103-023-03801-6 37310556

[B27] Dai J. Shen J. Chai Y. Chen H. (2021). IL-1 β impaired diabetic wound healing by regulating MMP-2 and MMP-9 through the p38 pathway. Mediat. Inflamm. 2021, 6645766. 10.1155/2021/6645766 34054346 PMC8149221

[B28] Damante C. A. De Micheli G. Miyagi S. P. H. Feist I. S. Marques M. M. (2009). Effect of laser phototherapy on the release of fibroblast growth factors by human gingival fibroblasts. Lasers Med. Sci. 24 (6), 885–891. 10.1007/s10103-008-0582-y 18600291

[B29] Dang Y. Ye X. Weng Y. Tong Z. Ren Q. (2010). Effects of the 532-nm and 1,064-nm Q-switched Nd:YAG lasers on collagen turnover of cultured human skin fibroblasts: a comparative study. Lasers Med. Sci. 25 (5), 719–726. 10.1007/s10103-009-0657-4 20490593

[B30] Daniell M. D. Hill J. S. (1991). A history of photodynamic therapy. Aust. N. Z. J. Surg. 61 (5), 340–348. 10.1111/J.1445-2197.1991.TB00230.X 2025186

[B31] Darby I. A. Desmoulière A. (2020). Scar formation: cellular mechanisms. Textb. Scar Manag., 19–26. 10.1007/978-3-030-44766-3_3 36351123

[B32] Darby I. A. Laverdet B. Bonté F. Desmoulière A. (2014). Fibroblasts and myofibroblasts in wound healing. Clin. Cosmet. Investigational Dermatology 7, 301–311. 10.2147/CCID.S50046 25395868 PMC4226391

[B33] Davidson S. Coles M. Thomas T. Kollias G. Ludewig B. Turley S. (2021). Fibroblasts as immune regulators in infection, inflammation and cancer. Nat. Rev. Immunol. 2021 21 (11), 704–717. 10.1038/s41577-021-00540-z 33911232

[B34] Demaria M. Ohtani N. Youssef S. Rodier F. Toussaint W. Mitchell J. (2014). An essential role for senescent cells in optimal wound healing through secretion of PDGF-AA. Dev. Cell 31 (6), 722–733. 10.1016/J.DEVCEL.2014.11.012 25499914 PMC4349629

[B35] Diller R. B. Tabor A. J. (2022). The role of the extracellular matrix (ECM) in wound healing: a review. Biomimetics 7 (3), 87. 10.3390/BIOMIMETICS7030087 35892357 PMC9326521

[B36] Diogo M. L. G. Campos T. M. Fonseca E. S. R. Pavani C. Horliana A. C. R. T. Fernandes K. P. S. (2021). Effect of blue light on acne vulgaris: a systematic review. Sensors 21 (20), 6943–13. 10.3390/s21206943 34696155 PMC8537635

[B37] dos Santos Cardoso F. dos Santos J. C. C. Gonzalez-Lima F. Araújo B. H. S. Lopes-Martins R. Á. B. Gomes da Silva S. (2021). Effects of chronic photobiomodulation with transcranial near-infrared laser on brain metabolomics of young and aged rats. Mol. Neurobiol. 58 (5), 2256–2268. 10.1007/s12035-020-02247-z 33417219

[B38] Esmaeelinejad M. Bayat M. (2013). Effect of low-level laser therapy on the release of interleukin-6 and basic fibroblast growth factor from cultured human skin fibroblasts in normal and high glucose mediums. J. Cosmet. Laser Ther. 15 (6), 310–317. 10.3109/14764172.2013.803366 23656570

[B39] Galiano R. D. Michaels, V J. Dobryansky M. Levine J. P. Gurtner G. C. (2004). Quantitative and reproducible murine model of excisional wound healing. Wound Repair Regen. 12 (4), 485–492. 10.1111/J.1067-1927.2004.12404.X 15260814

[B40] Garza Z. C. F. Born M. Hilbers P. van Riel N. Liebmann J. (2017). Visible blue light therapy: molecular mechanisms and therapeutic opportunities. Curr. Med. Chem. 25 (40), 5564–5577. 10.2174/0929867324666170727112206 28748760

[B41] Golovynska I. Golovynskyi S. Stepanov Y. V. Stepanova L. I. Qu J. Ohulchanskyy T. Y. (2021). Red and near-infrared light evokes Ca2+ influx, endoplasmic reticulum release and membrane depolarization in neurons and cancer cells. J. Photochem. Photobiol. B Biol. 214, 112088. 10.1016/j.jphotobiol.2020.112088 33278762

[B42] Gonzalez-Lima F. Rojas F. (2011). Low-level light therapy of the eye and brain. Eye Brain 3, 49–67. 10.2147/eb.s21391 28539775 PMC5436183

[B43] Grzybowski A. Pietrzak K. (2012). From patient to discoverer-Niels ryberg finsen (1860-1904)-the founder of phototherapy in dermatology. Clin. Dermatology 30 (4), 451–455. 10.1016/j.clindermatol.2011.11.019 22855977

[B44] Gu Q. Wang L. Huang F. Schwarz W. (2012). Stimulation of TRPV1 by green laser light. Evidence-based Complementary Altern. Med. 2012, 1–8. 10.1155/2012/857123 23365602 PMC3539758

[B45] Gurtner G. C. (2011). Surgical approaches to create murine models of human wound healing. J. Biomed. Biotechnol., 2011. 10.1155/2011/969618 PMC2995912 21151647

[B46] Hamblin M. R. (2016). Photobiomodulation or low-level laser therapy. J. Biophot. 9 (11–12), 1122–1124. 10.1002/jbio.201670113 27973730 PMC5215795

[B47] Hamblin M. R. Liebert A. (2022). Photobiomodulation therapy mechanisms beyond cytochrome c oxidase. Photobiomodul. Photomed. Laser Surg. 40 (2), 75–77. 10.1089/PHOTOB.2021.0119 34818111

[B48] Hernández-Bule M. L. Naharro-Rodríguez J. Bacci S. Fernández-Guarino M. (2024). Unlocking the power of light on the skin: a comprehensive review on photobiomodulation. Int. J. Mol. Sci. 25 (8), 4483. 10.3390/IJMS25084483 38674067 PMC11049838

[B49] Hong J. Y. Han H. S. Youn J. H. Kim H. Ryu H. Park K. Y. (2022). Irradiation with 590-nm yellow light-emitting diode light attenuates oxidative stress and modulates UVB-Induced change of dermal fibroblasts. Exp. Dermatol. 31 (6), 931–935. 10.1111/EXD.14542 35181944

[B50] Houreld N. N. Abrahamse H. (2008). Laser light influences cellular viability and proliferation in diabetic-wounded fibroblast cells in a dose- and wavelength-dependent manner. Lasers Med. Sci. 23 (1), 11–18. 10.1007/s10103-007-0445-y 17361392

[B51] Houreld N. N. Ayuk S. M. Abrahamse H. (2014). Expression of genes in normal fibroblast cells (WS1) in response to irradiation at 660 nm. J. Photochem. Photobiol. B Biol. 130, 146–152. 10.1016/j.jphotobiol.2013.11.018 24333762

[B52] Houreld N. N. Ayuk S. M. Abrahamse H. (2018). Cell adhesion molecules are mediated by photobiomodulation at 660 nm in diabetic wounded fibroblast cells. Cells 7 (4), 30–17. 10.3390/cells7040030 29659538 PMC5946107

[B53] Hsieh H. C. Tseng W. W. Wei A. C. (2022). Mathematical model of photobiomodulation on cytochrome c oxidase. Proc. - IEEE 22nd Int. Conf. Bioinforma. Bioeng. 2022, 187–192. 10.1109/BIBE55377.2022.00048

[B54] Huang Y. Y. Chen A. C. H. Carroll J. D. Hamblin M. R. (2009). Biphasic dose response in low level light therapy. Dose-Response 7 (4), 358–383. 10.2203/dose-response.09-027.Hamblin 20011653 PMC2790317

[B55] Jere S. W. Abrahamse H. Houreld N. N. (2017). The JAK/STAT signaling pathway and photobiomodulation in chronic wound healing. Cytokine Growth Factor Rev. 38 (October), 73–79. 10.1016/j.cytogfr.2017.10.001 29032938

[B56] Jere S. W. Houreld N. N. Abrahamse H. (2018). Photobiomodulation at 660 nm stimulates proliferation and migration of diabetic wounded cells *via* the expression of epidermal growth factor and the JAK/STAT pathway. J. Photochem. Photobiol. B Biol. 179, 74–83. 10.1016/j.jphotobiol.2017.12.026 29353701

[B57] Jere S. W. Houreld N. N. Abrahamse H. (2020). Photobiomodulation and the expression of genes related to the JAK/STAT signalling pathway in wounded and diabetic wounded cells. J. Photochem. Photobiol. B Biol. 204, 111791. 10.1016/j.jphotobiol.2020.111791 31981991

[B58] Jere S. W. Houreld N. N. Abrahamse H. (2022). Photobiomodulation activates the PI3K/AKT pathway in diabetic fibroblast cells *in vitro* . J. Photochem. Photobiol. B Biol. 237 (September), 112590. 10.1016/j.jphotobiol.2022.112590 36334508

[B59] Karkada G. Maiya G. A. Arany P. Rao M. Adiga S. Kamath S. U. (2022). Effect of photobiomodulation therapy on oxidative stress markers in healing dynamics of diabetic neuropathic wounds in wistar rats. Cell Biochem. Biophysics 80 (1), 151–160. 10.1007/S12013-021-01021-9 34331219 PMC8881248

[B60] Karu T. (1999). Primary and secondary mechanisms of action of visible to near-IR radiation on cells. J. Photochem. Photobiol. B Biol. 49 (1), 1–17. 10.1016/S1011-1344(98)00219-X 10365442

[B61] Karu T. (2000). Mechanisms of low-power laser light action on cellular level. Lasers Med. Dent. Rijeka Croat. Vitgraf 4159, 1–17. 10.1117/12.405918

[B62] Kasowanjete P. Houreld N. N. Abrahamse H. (2022). The effect of photomodulation on fibroblast growth factor and the Ras/MAPK signalling pathway: a review. J. Wound Care 31 (10), 832–845. 10.12968/jowc.2022.31.10.832 36240795

[B63] Kasowanjete P. Abrahamse H. Houreld N. N. (2023). Photobiomodulation at 660 nm stimulates *in vitro* diabetic wound healing *via* the Ras/MAPK Pathway. Cells 12 (7), 1080. 10.3390/cells12071080 37048153 PMC10093328

[B64] Keshri G. K. Kumar G. Sharma M. Bora K. Kumar B. Gupta A. (2021). Photobiomodulation effects of pulsed-NIR laser (810 nm) and LED (808 ± 3 nm) with identical treatment regimen on burn wound healing: a quantitative label-free global proteomic approach. J. Photochem. Photobiol. 6 (November 2020), 100024. 10.1016/j.jpap.2021.100024

[B65] Khan I. Rahman S. U. Tang E. Engel K. Hall B. Kulkarni A. B. (2021). Accelerated burn wound healing with photobiomodulation therapy involves activation of endogenous latent TGF-β1. Sci. Rep. 11 (1), 13371–15. 10.1038/s41598-021-92650-w 34183697 PMC8238984

[B66] Khoury J. G. Goldman M. P. (2008). Use of light-emitting diode photomodulation to reduce erythema and discomfort after intense pulsed light treatment of photodamage. J. Cosmet. Dermatology 7 (1), 30–34. 10.1111/j.1473-2165.2008.00358.x 18254808

[B67] Kolimi P. Narala S. Nyavanandi D. Youssef A. A. A. Dudhipala N. (2022). Innovative treatment strategies to accelerate wound healing: trajectory and recent advancements. Cells 11 (15), 2439. 10.3390/cells11152439 35954282 PMC9367945

[B68] Kreslavski V. D. Fomina I. R. Los D. A. Carpentier R. Kuznetsov V. V. Allakhverdiev S. I. (2012). Red and near infra-red signaling: hypothesis and perspectives. J. Photochem. Photobiol. C Photochem. Rev. 13 (3), 190–203. 10.1016/j.jphotochemrev.2012.01.002

[B69] Kriete A. Eils R. (2005). Computational systems biology. Comput. Syst. Biol. 420 (November), 1–409. 10.1016/B978-0-12-088786-6.X5019-9

[B70] Kuffler D. P. (2016). Photobiomodulation in promoting wound healing: a review. Regen. Med. 11 (1), 107–122. 10.2217/rme.15.82 26681143

[B71] Kumari J. Das K. Babaei M. Rokni G. R. Goldust M. (2023). The impact of blue light and digital screens on the skin. J. Cosmet. Dermatology 22 (4), 1185–1190. 10.1111/jocd.15576 36594795

[B72] Lanzafame R. (2020). Light dosing and tissue penetration: it is complicated. Photobiomodulation, Photomed. Laser Surg. 38 (7), 393–394. 10.1089/photob.2020.4843 32503388 PMC7374595

[B73] Lerman O. Z. Galiano R. D. Armour M. Levine J. P. Gurtner G. C. (2003). Cellular dysfunction in the diabetic fibroblast: impairment in migration, vascular endothelial growth factor production, and response to hypoxia. Am. J. Pathology 162 (1), 303–312. 10.1016/S0002-9440(10)63821-7 12507913 PMC1851127

[B74] Leyane T. S. Jere S. W. Houreld N. N. (2021). Cellular signalling and photobiomodulation in chronic wound repair. Int. J. Mol. Sci. 22 (20), 11223. 10.3390/ijms222011223 34681882 PMC8537491

[B75] Leyane T. S. Jere S. W. Houreld N. N. (2024). Effect of photobiomodulation at 830 nm on gene expression correlated with JAK/STAT signalling in wounded and diabetic wounded fibroblasts *in vitro* . J. Biophot. 17 (2), e202300230–e202300238. 10.1002/jbio.202300230 38010362

[B76] Li Z. Zhang C. Wang L. Zhang Q. Dong Y. Sha X. (2025). Chitooligosaccharides promote diabetic wound healing by mediating fibroblast proliferation and migration. Sci. Rep. 15 (1), 556–15. 10.1038/S41598-024-84398-W 39747336 PMC11697320

[B77] Lynch M. D. Watt F. M. (2018). Fibroblast heterogeneity: implications for human disease. J. Clin. Investigation 128 (1), 26–35. 10.1172/JCI93555 29293096 PMC5749540

[B78] Malthiery E. Chouaib B. Hernandez-Lopez A. M. Martin M. Gergely C. Torres J. H. (2021). Effects of green light photobiomodulation on dental pulp stem cells: enhanced proliferation and improved wound healing by cytoskeleton reorganization and cell softening. Lasers Med. Sci. 36 (2), 437–445. 10.1007/s10103-020-03092-1 32621128

[B79] Manning B. D. Cantley L. C. (2007). AKT/PKB signaling: navigating downstream. Cell 129 (7), 1261–1274. 10.1016/j.cell.2007.06.009 17604717 PMC2756685

[B80] Mathur R. K. Sahu K. Saraf S. Patheja P. Khan F. Gupta P. K. (2017). Low-level laser therapy as an adjunct to conventional therapy in the treatment of diabetic foot ulcers. Lasers Med. Sci. 32 (2), 275–282. 10.1007/s10103-016-2109-2 27896528

[B81] MgGuff P. E. Deterling R. A. Gottlieb L. S. (1965). Tumoricidal effect of laser energy on experimental and human malignant tumors. N. Engl. J. Med. 273 (9), 490–492. 10.1056/nejm196508262730906 5318702

[B82] Mgwenya T. N. Abrahamse H. Houreld N. N. (2024). Photobiomodulation studies on diabetic wound healing: an insight into the inflammatory pathway in diabetic wound healing. Wound Repair Regen. 33 (1), e13239. 10.1111/WRR.13239 39610015 PMC11628774

[B83] Migliario M. Sabbatini M. Mortellaro C. Renò F. (2018). Near infrared low-level laser therapy and cell proliferation: the emerging role of redox sensitive signal transduction pathways. J. Biophot. 11 (11), e201800025. 10.1002/jbio.201800025 29722183

[B84] Moco S. (2022). Studying metabolism by NMR-Based metabolomics. Front. Mol. Biosci. 9 (April), 882487–12. 10.3389/fmolb.2022.882487 35573745 PMC9094115

[B85] Mokoena D. R. Houreld N. N. Dhilip Kumar S. S. Abrahamse H. (2020). Photobiomodulation at 660 nm Stimulates Fibroblast Differentiation. Lasers Surg. Med. 52 (7), 671–681. 10.1002/lsm.23204 31820475

[B86] Monteiro M. M. Amorim dos Santos J. Paiva Barbosa V. Rezende T. M. B. Guerra E. N. S. (2024). Photobiomodulation effects on fibroblasts and keratinocytes after ionizing radiation and bacterial stimulus. Archives Oral Biol. 159, 105874. 10.1016/j.archoralbio.2023.105874 38147800

[B87] Nakayama E. Kushibiki T. Mayumi Y. Fushuku S. Nakamura T. Kiyosawa T. (2023). Optimal blue light irradiation conditions for the treatment of acne vulgaris in a mouse model. J. Photochem. Photobiol. B Biol. 239, 112651. 10.1016/J.JPHOTOBIOL.2023.112651 36680809

[B88] Nalbantoglu S. Karadag A. (2019). Introductory chapter: insight into the OMICS technologies and molecular medicine. Mol. Med. 10.5772/INTECHOPEN.86450

[B89] Ogita M. Tsuchida S. Aoki A. Satoh M. Kado S. Sawabe M. (2015). Increased cell proliferation and differential protein expression induced by low-level Er:YAG laser irradiation in human gingival fibroblasts: proteomic analysis. Lasers Med. Sci. 30 (7), 1855–1866. 10.1007/s10103-014-1691-4 25429773

[B90] Oyebode O. A. Houreld N. N. (2022). Photobiomodulation at 830 nm Stimulates Migration, Survival and Proliferation of Fibroblast cells. Diabetes, Metabolic Syndrome Obes. 15, 2885–2900. 10.2147/DMSO.S374649 36172056 PMC9510698

[B91] Oyebode O. Houreld N. N. Abrahamse H. (2021). Photobiomodulation in diabetic wound healing: a review of red and near-infrared wavelength applications. Cell Biochem. Funct. 39 (5), 596–612. 10.1002/cbf.3629 33870502

[B92] Pakyari M. Farrokhi A. Maharlooei M. K. Ghahary A. (2013). Critical role of transforming growth factor beta in different phases of wound healing. Adv. Wound Care 2 (5), 215–224. 10.1089/wound.2012.0406 24527344 PMC3857353

[B93] Pchelin P. Shkarupa D. Smetanina N. Grigorieva T. Lapshin R. Schelchkova N. (2023). Journal of Photochemistry and Photobiology, B: biology red light photobiomodulation rescues murine brain mitochondrial respiration after acute hypobaric hypoxia. J. Photochem. and Photobiol. B Biol. 239 (December 2022), 112643. 10.1016/j.jphotobiol.2022.112643 36610350

[B94] Pieper C. Lee E. B. Swali R. Harp K. Wysong A. (2022). Effects of blue light on the skin and its therapeutic uses: photodynamic therapy and beyond. Dermatol. Surg. 48 (8), 802–808. 10.1097/DSS.0000000000003500 35917260

[B95] Plikus M. V. Wang X. Sinha S. Forte E. Thompson S. M. Herzog E. L. (2021). Fibroblasts: origins, definitions, and functions in health and disease. Cell 184 (15), 3852–3872. 10.1016/j.cell.2021.06.024 34297930 PMC8566693

[B96] Ponnusamy S. (2020). Photobiomodulation therapy in diabetic wound healing, Wound Healing, Tissue repair, and regeneration in diabetes. Elsevier Inc. 10.1016/B978-0-12-816413-6.00015-0

[B97] Pourang A. Tisack A. Ezekwe N. Torres A. E. Kohli I. Hamzavi I. H. (2022). Effects of visible light on mechanisms of skin photoaging. Photodermatol. Photoimmunol. Photomed. 38 (3), 191–196. 10.1111/phpp.12736 34585779

[B98] Priyadarshi A. Keshri G. K. Gupta A. (2023). Dual-NIR wavelength (pulsed 810 nm and superpulsed 904 nm lasers) photobiomodulation therapy synergistically augments full-thickness burn wound healing: a non-invasive approach. J. Photochem. Photobiol. B Biol. 246, 112761. 10.1016/J.JPHOTOBIOL.2023.112761 37542937

[B99] Purbhoo-Makan M. Houreld N. N. Enwemeka C. S. (2022). The effects of blue light on human fibroblasts and diabetic wound healing. Life 12 (9), 1431–20. 10.3390/life12091431 36143466 PMC9505688

[B100] Rahbar Layegh E. Fadaei Fathabadi F. Lotfinia M. Zare F. Mohammadi Tofigh A. Abrishami S. (2020). Photobiomodulation therapy improves the growth factor and cytokine secretory profile in human type 2 diabetic fibroblasts. J. Photochem. Photobiol. B Biol. 210, 111962. 10.1016/j.jphotobiol.2020.111962 32712344

[B101] Raizman R. Gavish L. (2020). At-Home self-applied photobiomodulation device for the treatment of diabetic foot ulcers in adults with type 2 diabetes: report of 4 cases. Can. J. Diabetes 44 (5), 375–378. 10.1016/j.jcjd.2020.01.010 32241752

[B102] Rajendran N. K. Houreld N. N. (2022). Photobiomodulation hastens diabetic wound healing *via* modulation of the PI3K/AKT/FoxO1 pathway in an adipose derived stem cell-fibroblast co-culture. J. Photochem. Photobiol. 12 (November), 100157. 10.1016/j.jpap.2022.100157

[B103] Rajendran N. K. Dhilip Kumar S. S. Houreld N. N. Abrahamse H. (2019). Understanding the perspectives of forkhead transcription factors in delayed wound healing. J. Cell Commun. Signal. 13 (2), 151–162. 10.1007/s12079-018-0484-0 30088222 PMC6498300

[B104] Rajendran N. K. Houreld N. N. Abrahamse H. (2021). Photobiomodulation reduces oxidative stress in diabetic wounded fibroblast cells by inhibiting the FOXO1 signaling pathway. J. Cell Commun. Signal. 15 (2), 195–206. 10.1007/s12079-020-00588-x 33052534 PMC7991023

[B105] Reyes A. Ortiz G. Duarte L. F. Fernández C. Hernández-Armengol R. Palacios P. A. (2023). Contribution of viral and bacterial infections to senescence and immunosenescence. Front. Cell. Infect. Microbiol. 13, 1229098. 10.3389/FCIMB.2023.1229098 37753486 PMC10518457

[B106] Roelandts R. (2002). The history of phototherapy: something new under the sun? J. Am. Acad. Dermatology 46 (6), 926–930. 10.1067/mjd.2002.121354 12063493

[B107] Ruggiero E. Alonso-de Castro S. Habtemariam A. Salassa L. (2016). Upconverting nanoparticles for the near infrared photoactivation of transition metal complexes: new opportunities and challenges in medicinal inorganic photochemistry. Dalton Trans. 45 (33), 13012–13020. 10.1039/C6DT01428C 27482656

[B108] Saied G. M. Kamel R. M. Labib A. M. Said M. T. Mohamed A. Z. (2011). The diabetic foot and leg: combined He-Ne and infrared low-intensity lasers improve skin blood perfusion and prevent potential complications. A prospective study on 30 Egyptian patients. Lasers Med. Sci. 26 (5), 627–632. 10.1007/s10103-011-0911-4 21455785

[B109] Samuel Enwemeka C. Monteiro F. Oliveira S. Costa I. Catarino S. O. Carvalho Ó. (2024). Optimization of a photobiomodulation protocol to improve the cell viability, proliferation and protein expression in osteoblasts and periodontal ligament fibroblasts for accelerated orthodontic treatment. Biomedicines 12 (1), 180. 10.3390/BIOMEDICINES12010180 38255285 PMC10813108

[B110] Saygun I. Karacay S. Serdar M. Ural A. U. Sencimen M. Kurtis B. (2008). Effects of laser irradiation on the release of basic fibroblast growth factor (bFGF), insulin like growth factor-1 (IGF-1), and receptor of IGF-1 (IGFBP3) from gingival fibroblasts. Lasers Med. Sci. 23 (2), 211–215. 10.1007/s10103-007-0477-3 17619941

[B111] Sequeira J. H. O’Donovan W. J. (1925). THE PRACTICE OF ARTIFICIAL LIGHT-BATH TREATMENT. Lancet 205 (5305), 909–912. 10.1016/S0140-6736(01)22259-4

[B112] Serrage H. Heiskanen V. Palin W. M. Cooper P. R. Milward M. R. Hadis M. (2019). Under the spotlight: mechanisms of photobiomodulation concentrating on blue and green light. Photochem. Photobiological Sci. 18 (8), 1877–1909. 10.1039/c9pp00089e 31183484 PMC6685747

[B113] Sexton D. J. Muruganandam A. McKenney D. J. Mutus B. (1994). VISIBLE LIGHT PHOTOCHEMICAL RELEASE OF NITRIC OXIDE FROM *S*‐NITROSOGLUTATHIONE: POTENTIAL PHOTOCHEMOTHERAPEUTIC APPLICATIONS. Photochem. Photobiol. 59 (4), 463–467. 10.1111/j.1751-1097.1994.tb05065.x 8022889

[B114] Sharma S. K. Kharkwal G. B. Sajo M. Huang Y. De Taboada L. McCarthy T. (2011). Dose response effects of 810 nm laser light on mouse primary cortical neurons. Lasers Surg. Med. 43 (8), 851–859. 10.1002/LSM.21100 21956634 PMC3199299

[B115] Shi Y. Massagué J. (2003). Mechanisms of TGF-β signaling from cell membrane to the nucleus. Cell 113 (6), 685–700. 10.1016/S0092-8674(03)00432-X 12809600

[B116] Sklar L. R. Almutawa F. Lim H. W. Hamzavi I. (2013). Effects of ultraviolet radiation, visible light, and infrared radiation on erythema and pigmentation: a review. Photochem. Photobiological Sci. 12 (1), 54–64. 10.1039/c2pp25152c 23111621

[B117] Sorrell J. M. Caplan A. I. (2004). Fibroblast heterogeneity: more than skin deep. J. Cell Sci. 117 (5), 667–675. 10.1242/jcs.01005 14754903

[B118] Suh S. Choi E. H. Atanaskova Mesinkovska N. (2020). The expression of opsins in the human skin and its implications for photobiomodulation: a systematic review. Photodermatol. Photoimmunol. Photomed. 36 (5), 329–338. 10.1111/phpp.12578 32431001 PMC7674233

[B119] Takeuchi M. Nishisho T. Toki S. Kawaguchi S. Tamaki S. Oya T. (2023). Blue light induces apoptosis and autophagy by promoting ROS-Mediated mitochondrial dysfunction in synovial sarcoma. Cancer Med. 12 (8), 9668–9683. 10.1002/CAM4.5664 36722116 PMC10166932

[B120] Talbott H. E. Mascharak S. Griffin M. Wan D. C. Longaker M. T. (2022). Wound healing, fibroblast heterogeneity, and fibrosis. Cell stem cell 29 (8), 1161–1180. 10.1016/J.STEM.2022.07.006 35931028 PMC9357250

[B121] Terakita A. Nagata T. (2014). Functional properties of opsins and their contribution to light-sensing physiology. Physiology 31 (10), 653–659. 10.2108/ZS140094 25284384

[B122] Thomson S. E. McLennan S. V. Twigg S. M. (2006). Growth factors in diabetic complications. Expert Rev. Clin. Immunol. 2 (3), 403–418. 10.1586/1744666X.2.3.403 20476912

[B123] Tracy L. E. Minasian R. A. Caterson E. J. (2016). Extracellular Matrix and dermal fibroblast function in the healing wound. Adv. Wound Care 5 (3), 119–136. 10.1089/WOUND.2014.0561 26989578 PMC4779293

[B124] Tripodi N. Feehan J. Husaric M. Kiatos D. Sidiroglou F. Fraser S. (2020). Good, better, best? The effects of polarization on photobiomodulation therapy. J. Biophot. 13 (5), e201960230. 10.1002/JBIO.201960230 32077232

[B125] Tripodi N. Corcoran D. Antonello P. Balic N. Caddy D. Knight A. (2021). The effects of photobiomodulation on human dermal fibroblasts *in vitro:* a systematic review. J. Photochem. Photobiol. B Biol. 214, 112100. 10.1016/j.jphotobiol.2020.112100 33316625

[B126] Tripodi N. Sidiroglou F. Apostolopoulos V. Feehan J. (2023). Transcriptome analysis of the effects of polarized photobiomodulation on human dermal fibroblasts. J. Photochem. Photobiol. B Biol. 242 (March), 112696. 10.1016/j.jphotobiol.2023.112696 36958088

[B127] Van Tran V. Chae M. Moon J. Y. Lee Y. C. (2021). Light emitting diodes technology-based photobiomodulation therapy (PBMT) for dermatology and aesthetics: recent applications, challenges, and perspectives. Opt. Laser Technol. 135 (March 2020), 106698. 10.1016/j.optlastec.2020.106698

[B128] Voza F. A. Huerta C. T. Le N. Shao H. Ribieras A. Ortiz Y. (2024). Fibroblasts in diabetic foot ulcers. Int. J. Mol. Sci. 25 (4), 2172–24. 10.3390/ijms25042172 38396848 PMC10889208

[B129] Wall I. B. Moseley R. Baird D. M. Kipling D. Giles P. Laffafian I. (2008). Fibroblast dysfunction is a key factor in the non-healing of chronic venous leg ulcers. J. Investigative Dermatology 128 (10), 2526–2540. 10.1038/jid.2008.114 18449211

[B130] Wang Z. Shi C. (2020). Cellular senescence is a promising target for chronic wounds: a comprehensive review. Burns and Trauma 8, tkaa021. 10.1093/BURNST/TKAA021 32607375 PMC7309580

[B131] Wang L. Zhang D. Schwarz W. (2014). TRPV channels in mast cells as a target for low-level-laser therapy. Cells 2014 3 (3), 662–673. 10.3390/CELLS3030662 24971848 PMC4197630

[B132] Wang Y. Huang Y. Y. Lyu P. Hamblin M. R. (2017). Red (660 nm) or near-infrared (810 nm) photobiomodulation stimulates, while blue (415 nm), green (540 nm) light inhibits proliferation in human adipose-derived stem cells. Sci. Rep. 7 (1), 7781–10. 10.1038/s41598-017-07525-w 28798481 PMC5552860

[B133] Wang B. Han J. Elisseeff J. H. Demaria M. (2024). The senescence-associated secretory phenotype and its physiological and pathological implications. Nat. Rev. Mol. Cell Biol. 25 (12), 958–978. 10.1038/S41580-024-00727-X 38654098

[B134] Wilkinson H. N. Hardman M. J. (2020). Senescence in wound repair: emerging strategies to target chronic healing wounds. Front. Cell Dev. Biol. 8, 773. 10.3389/fcell.2020.00773 32850866 PMC7431694

[B135] Wilkinson H. N. Hardman M. J. (2023). Wound healing: cellular mechanisms and pathological outcomes. Adv. Surg. Med. Specialties 10, 200223–200370. 10.1098/rsob.200223 32993416 PMC7536089

[B136] Yang M. Y. Chang C. J. Chen L. Y. (2017). Blue light induced reactive oxygen species from flavin mononucleotide and flavin adenine dinucleotide on lethality of HeLa cells. J. Photochem. Photobiol. B Biol. 173, 325–332. 10.1016/j.jphotobiol.2017.06.014 28633062

[B137] Yu J. Choi S. Um J. Park K. S. (2017). Reduced expression of YAP in dermal fibroblasts is associated with impaired wound healing in type 2 diabetic mice. Tissue Eng. Regen. Med. 14 (1), 49–55. 10.1007/s13770-016-0019-9 30603461 PMC6171570

[B138] Zein R. Selting W. Hamblin M. R. (2018). Review of light parameters and photobiomodulation efficacy: dive into complexity. J. Biomed. Opt. 23 (12), 1. 10.1117/1.jbo.23.12.120901 30550048 PMC8355782

[B139] Zhang Y. Fong C. C. Tsang C. H. Yang Z. Yang M. (2003). cDNA microarray analysis of gene expression profiles in human fibroblast cells irradiated with red light. J. Investigative Dermatology 120 (5), 849–857. 10.1046/j.1523-1747.2003.12133.x 12713592

[B140] Zhang L. Xing D. Gao X. Wu S. (2009). Low-power laser irradiation promotes cell proliferation by activating PI3K/Akt pathway. J. Cell. Physiology 219 (3), 553–562. 10.1002/jcp.21697 19142866

[B141] Zhong J. Hu N. Xiong X. Lei Q. Li L. (2011). A novel promising therapy for skin aging: dermal multipotent stem cells against photoaged skin by activation of TGF-β/Smad and p38 MAPK signaling pathway. Med. Hypotheses 76, 343–346. 10.1016/j.mehy.2010.10.035 21093160

